# Control of *Helicobacter pylori* with engineered probiotics secreting selective guided antimicrobial peptides

**DOI:** 10.1128/spectrum.02014-23

**Published:** 2023-09-15

**Authors:** Ankan Choudhury, Patrick S. Ortiz, Mikaeel Young, Md. Toslim Mahmud, Ryan T. Stoffel, K. Leigh Greathouse, Christopher M. Kearney

**Affiliations:** 1 Department of Biology, Baylor University, Waco, Texas, USA; 2 Baylor Sciences Building Vivarium, Baylor University, Waco, Texas, USA; 3 Robbins College of Health and Human Sciences, Baylor University, Waco, Texas, USA; University of California, San Diego, La Jolla, California, USA

**Keywords:** *Helicobacter pylori*, selective, antimicrobial, probiotic, microbiome, dysbiosis, mouse

## Abstract

*Helicobacter pylori* is the primary cause of 78% of gastric cancer cases, providing an opportunity to prevent cancer by controlling a single bacterial pathogen within the complex gastric microbiota. We developed highly selective antimicrobial agents against *H. pylori* by fusing an *H. pylori*-binding guide peptide (MM1) to broad-spectrum antimicrobial peptides. The common dairy probiotic *Lactococcus lactis* was then engineered to secrete these guided antimicrobial peptides (gAMPs). When co-cultured *in vitro* with *H. pylori*, the gAMP probiotics lost no toxicity compared to unguided AMP probiotics against the target, *H. pylori*, while losing >90% of their toxicity against two tested off-target bacteria. To test binding to *H. pylori*, the MM1 guide was fused to green fluorescent protein (GFP), resulting in enhanced binding compared to unguided GFP as measured by flow cytometry. In contrast, MM1-GFP showed no increased binding over GFP against five different off-target bacteria. These highly selective gAMP probiotics were then tested by oral gavage in mice infected with *H. pylori*. As a therapy, the probiotics outperformed antibiotic treatment, effectively eliminating *H. pylori* in just 5 days, and also protected mice from challenge infection as a prophylactic. As expected, the gAMP probiotics were as toxic against *H. pylori* as the unguided AMP probiotics. However, a strong rebound in gastric species diversity was found with both the selective gAMP probiotics and the non-selective AMP probiotics. Eliminating the extreme microbial dysbiosis caused by *H. pylori* appeared to be the major factor in diversity recovery.

**Importance:**

Alternatives to antibiotics in the control of *Helicobacter pylori* and the prevention of gastric cancer are needed. The high prevalence of *H. pylori* in the human population, the induction of microbial dysbiosis by antibiotics, and increasing antibiotic resistance call for a more sustainable approach. By selectively eliminating the pathogen and retaining the commensal community, *H. pylori* control may be achieved without adverse health outcomes. Antibiotics are typically used as a therapeutic post-infection, but a more targeted, less disruptive approach could be used as a long-term prophylactic against *H. pylori* or, by extension, against other gastrointestinal pathogens. Furthermore, the modular nature of the guided antimicrobial peptide (gAMP) technology allows for the substitution of different guides for different pathogens and the use of a cocktail of gAMPs to avoid the development of pathogen resistance.

## INTRODUCTION

Connections between bacterial infection and carcinogenesis are beginning to emerge (1, 2). However, for most cancers being examined, contributions by multiple bacterial taxa and various host and abiotic factors make drawing these connections complex and finding a single control strategy difficult (3). However, gastric cancer caused by *Helicobacter pylori* stands out as an ideal system for a “single target” antibacterial strategy to prevent cancer development. *H. pylori* is classified as a Group I carcinogen (4) and is estimated to be the root cause of 78% or more of gastric cancer cases (5 – 7). Gastric cancer is the third most deadly cancer globally, claiming over 750,000 lives in 2020, mostly in East Asia (8). Gastric cancer prevention has been deemed addressable by regional antibiotic programs (9, 10), and there are recommendations for national-level *H. pylori* screening and eradication campaigns using multi-antibiotic treatments since *H. pylori* is found in approximately 43% of the human population (6). The rationale for this large-scale approach lies in halting *H. pylori*-induced inflammation at the earliest stage of the Correa cascade, which progresses from normal mucosa infected with *H. pylori* to atrophic gastritis to inflammation-induced hyperplasia to adenocarcinoma (11).

However, large-scale antibiotic treatment programs create unnecessary risks of increasing antibiotic resistance (12 – 14) and microbial dysbiosis (15, 16). High resistance frequencies are now seen in East Asia, with 17% for clarithromycin, 44% for metronidazole, and 18% for levofloxacin (17), which makes the standard multi-antibiotic treatment against *H. pylori* problematic. Eradication rates using multi-antibiotic treatments are lower than 80% in most published trials (6). Proper antibiotic stewardship can address this by using gastric biopsies and *H. pylori* culturing to establish the antibiotic resistance profile for a region, allowing recommendations for an empiric antibiotic combination to increase effectiveness (18). However, in low-resource regions, antibiotics are often available without a prescription, encouraging patient self-dosing, and the cost of *H. pylori* screening, antibiotic profiling, physician visits, and the optimal medication combination may not be seen as fundable by local governments. Another problem with antibiotics is their effect on the native microbiota. Although some studies (19, 20) have demonstrated an eventual return to normal gastrointestinal diversity after antibiotic treatment for the elimination of *H. pylori*, others have uncovered shifts in microbial composition (21, 22). The effect of “flattening” the microbiota composition of a large and varied human population during a widespread antibiotic campaign may lead to opportunities for colonization by other pathogens as well as other detrimental health effects (23, 24). Hence, we propose a novel and more sustainable approach by creating precision antimicrobials specifically targeting *H. pylori*.

**Fig 1:**
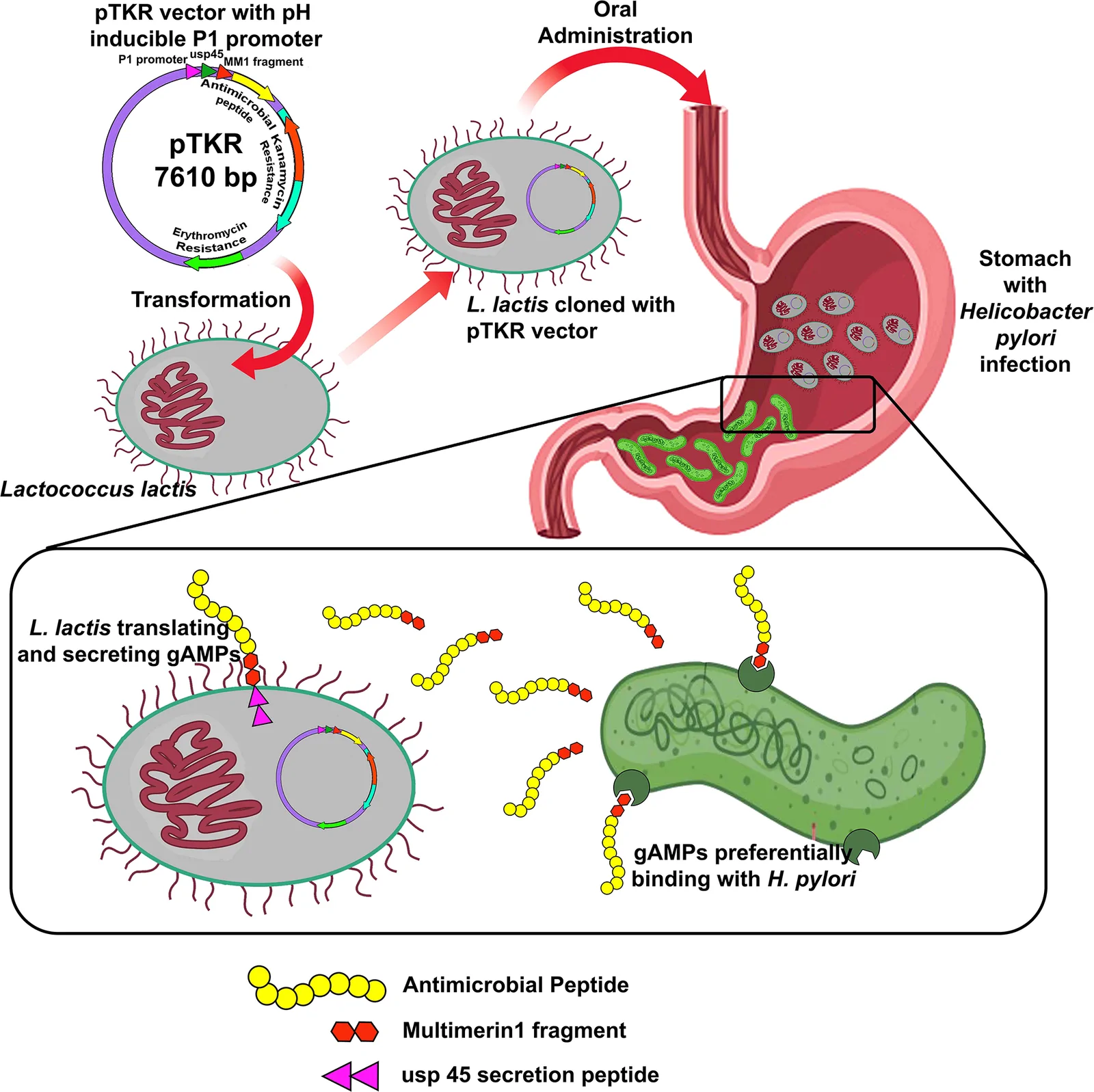
Precision targeting of *H. pylori* using probiotic delivery of guided antimicrobial peptides (gAMPs). The probiotic *Lactococcus lactis* carries the *Escherichia coli*/*L. lactis* shuttle vector, pTKR, for the expression of gAMPs in the mouse stomach, allowing rapid engineering in *E. coli* and transfer to *L. lactis*. The gAMPs were placed downstream of the acid-inducible P1 promoter and the usp secretion signal peptide to allow secretion in the acidic stomach environment. The guide attached to the N-terminus of the AMP was that portion of the human thrombin protein, Multimerin-1, that binds to *H. pylori*.

In the present study, we fused an antimicrobial peptide (AMP) to a guide peptide that specifically binds to *H. pylori* cells to create a precision antimicrobial that specifically targets *H. pylori* (Fig. 1). The guide peptide specifically binds to VacA (25), a secreted virulence factor that is also found on the surface of *H. pylori* (26). The *vacA* gene has been detected in all examined isolates, and its expression is only rarely absent (27). There are significant clinical correlations between *H. pylori*-induced cancer and the presence of its two major virulence factors, VacA and CagA (28, 29). Furthermore, the carcinogenicity of strains possessing virulent variants of these factors has been directly demonstrated in the Mongolian gerbil gastric cancer model (30). The 20-amino acid guide peptide (MM-1: MQKMTDQVNYQAMKLTLLQK) used in this study comprises the sequence from the human receptor protein, Multimerin-1, which binds to the *H. pylori* virulence factor, VacA (25).

Keeping in mind the potential of future clinical applications, we next considered how to avoid the cost of an expensive purified peptide drug and degradation of the peptide in the stomach. To address this, we delivered the guided antimicrobial peptides (gAMPs) via the food-grade probiotic *Lactococcus lactis*, which is commonly used in the dairy industry (31). *L. lactis* was chosen as it is the first Generally Regarded As Safe organism that the FDA has authorized to be used as a therapeutic agent to deliver heterologous protein drugs in humans (32, 33). The probiotic was engineered using a plasmid pT1NX (34 – 37), which uses the usp45 secretion signal peptide inherent to *L. lactis* (38), and is expressed by the P1 promoter (39), which is induced by a low pH such as that found in the stomach. We further modified pT1NX to contain a kanamycin resistance cassette *kanR* and an *Escherichia coli* origin of replication cassette *oriC*, which transformed pT1NX into a convenient shuttle vector (“pTRK”; Fig. 1) suitable for being cloned into both *L. lactis* and *E. coli*.

These engineered probiotics secreting AMPs or gAMPs surpassed antibiotics as therapeutics or prophylactics in a mouse model of *H. pylori* infection, effectively eliminating the infection in only 5 days after a single dose of orally administered probiotic. The probiotics also outperformed the antibiotic treatments by promoting rapid recovery from dysbiosis in mice, as measured by an internal microbial dysbiosis index. Our preliminary *in vitro* tests clearly showed a drastic attenuation of the toxicity of the gAMPs against off-target bacteria as compared to unguided AMPs. It was expected, therefore, that in the mouse model, these highly selective gAMPs would promote greater microbial diversity than unguided AMPs in the recovery of the infected gastric microbiota following therapy. However, a strong and rapid recovery of diversity was seen with both AMP and gAMP probiotics. Apparently, the rapid elimination of *H. pylori*-induced dysbiosis was the crucial factor in the rapid recovery of diversity.

## RESULTS

### Guided GFP protein selectively binds to *H. pylori* cells

**Fig 2:**
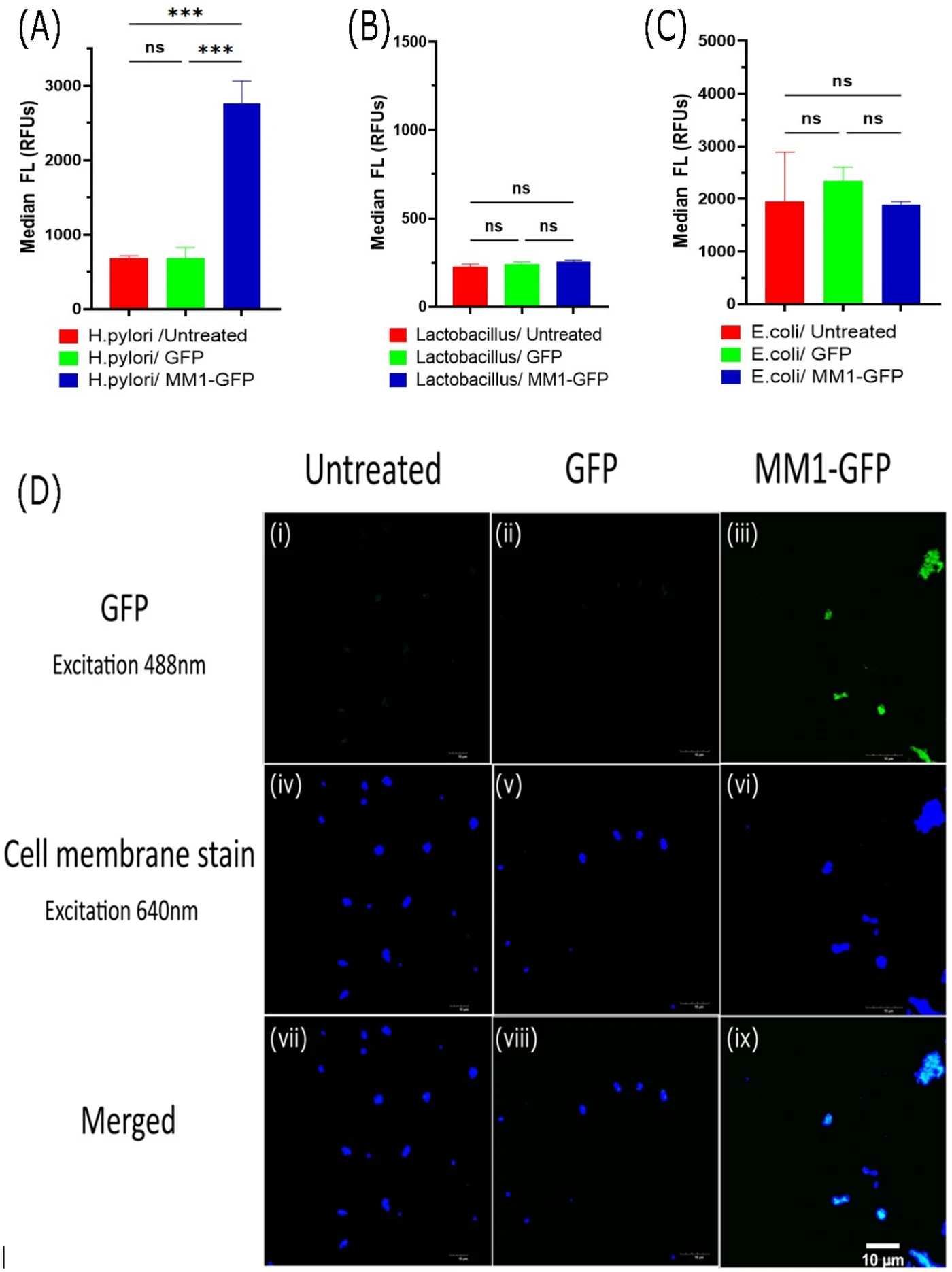
MM1-guided GFP (MM1-GFP) protein binds specifically to *H. pylori* cells. (**A**) Protein preparations of MM1-GFP, but not GFP, bound to *H. pylori* cells. Fluorescence intensity of cells of target bacterium *H. pylori* 60,190 WT untreated (red) or treated with GFP (green) or MM1-GFP (blue) with averaged median fluorescence (*n* = 3) in relative fluorescence units (RFUs) obtained from BD FACSverse flow cytometer using blue 488_nm_ laser and a 488/10 bandpass filter; standard deviation shown; statistical significance (one-way analysis of variance, one-way ANOVA; ns, not significant; ***P ≤ 0.001). (B and C) Neither MM1-GFP nor GFP protein significantly bound to off-target bacterial cells. Flow cytometry as in (**A**) for the cells of off-target bacteria *Lactobacillus plantarum* (**B**) or *Escherichia coli* K12 (**C**). (**D**) Confocal microscopy demonstrated that MM1-GFP, but not GFP, bound strongly to *Helicobacter pylori* cells. Imaging of *H. pylori* 60,190 WT cells untreated (first column) or treated with GFP (second column) or MM1-GFP (third column). Top row: visualization of GFP or MM1-GFP fluorescence at 488 nm. Middle row: visualization of bacterial cells (CellBrite stain, 640 nm). Bottom row: merged images.

To verify that the MM1 guide binds *H. pylori* cells selectively, the MM1 sequence and linker peptide were fused to green fluorescent protein (GFP), creating MM1-GFP. MM1-GFP binding to *H. pylori* was quantified using flow cytometry. In Fig. 2A, flow cytometry revealed significant binding of MM1-GFP (dark blue bar) to *H. pylori* that was 4.1 times stronger than unmodified GFP, while unmodified GFP fluorescence did not differ from the untreated control. In contrast, no fluorescence was seen above the untreated control for MM1-GFP incubated with the off-target bacteria, *Lactobacillus plantarum* or *E. coli K-12* (Fig. 2B and C). Thus, MM1-GFP binds selectively to *H. pylori* cells.

To visualize the binding of MM1-GFP to *H. pylori* cells and to confirm the flow cytometry results, confocal microscopy was used to image *H. pylori* cells bound to GFP or MM1-GFP (Fig. 2D). Binding of *H. pylori* cells was visualized by staining with a cell membrane dye (Cell Brite 640; excitation 640 nm) to visualize cells and excitation at 488 nm to visualize bound GFP. The merged images (Fig. 2D) clearly show co-localization of MM1-GFP and *H. pylori* cells, but no binding of GFP to *H. pylori* cells, indicating selective binding by the MM1 guide.

To investigate if MM1 binds with *H. pylori* proteins other than VacA, we tested the binding of MM1-guided GFP against both wild-type 60190 *H. pylori* bacteria and an isogenic mutant in which a kanamycin resistance cassette was inserted into the VacA ORF to disrupt expression. This mutant, 60190:v1, was a generous gift from Dr. Timothy Cover and had been previously tested for lack of VacA expression by Western blot and loss of cytotoxicity phenotype (40). Surprisingly, this 60190:v1 mutant displayed a binding phenotype similar to the wild type; that is, it showed increased MM1-GFP binding to the bacterial cells compared to unguided GFP (data not shown). This would imply that, while the MM1 guide targets *H. pylori* specifically, this guide binds to another surface target(s) as well as VacA. To further verify these results, we created our own VacA knockout mutant (vacA-4S) by introducing four stop codons at amino acid positions 4, 5, 23, and 27 of the *vacA* ORF. This vacA-4s 60190 *H.* *pylori* behaved similarly to the 60190 wild type and the 60190:v1 mutant, with strong, significantly greater binding by guided GFP compared to unguided GFP (Fig. S1), supporting the idea of an alternate binding site for the MM1 guide besides VacA.

Regardless of the specific identity of the *H. pylori* target(s) in MM1, we found that the targeting was quite specific for *H. pylori*. Three additional off-target bacteria were analyzed using flow cytometry to test the specificity of MM1 guided GFP. *Pseudomonas aeruginosa*, *Staphylococcus aureus*, and *Alcaligenes faecalis* were analyzed for binding to MM1-GFP, GFP, and the buffer control. No differences in binding between GFP and MM1-GFP were observed, further indicating that the MM1 guide, regardless of the presence or absence of VacA, specifically binds only *H. pylori* (Fig. S2).

### Guided AMP probiotics selectively kill *H*. *pylori* when co-cultured *in vitro*

Engineered probiotic lines were generated that express AMPs or MM1-gAMPs for testing against *H. pylori* or off-target bacteria by *in vitro* co-culture. Three AMPs were tested (alyteserin, laterosporulin, and CRAMP; Table S1) to determine if the effect of the guide peptide was consistent across different AMPs. The three AMPs are more strongly active against Gram (−) bacteria (including *H. pylori*) than Gram (+). The MM1 guide peptide and a linker peptide were fused to the N-terminus of each AMP to create the three gAMPs. A shuttle vector, pTKR (Fig. S1) was created, which allowed for rapid engineering in *E. coli* and transfer of the engineered plasmid to the probiotic *Lactococcus lactis*. The AMP and gAMP ORFs were placed downstream of the acid-inducible promoter, P1, in pTKR (Fig. S1) to allow for expression *in vitro* (via natural lactic acid production by *L. lactis*) and in the acidic environment of the stomach. In this way, a rapid system for testing gAMP probiotics *in vitro* or *in vivo* was developed.

**Fig 3:**
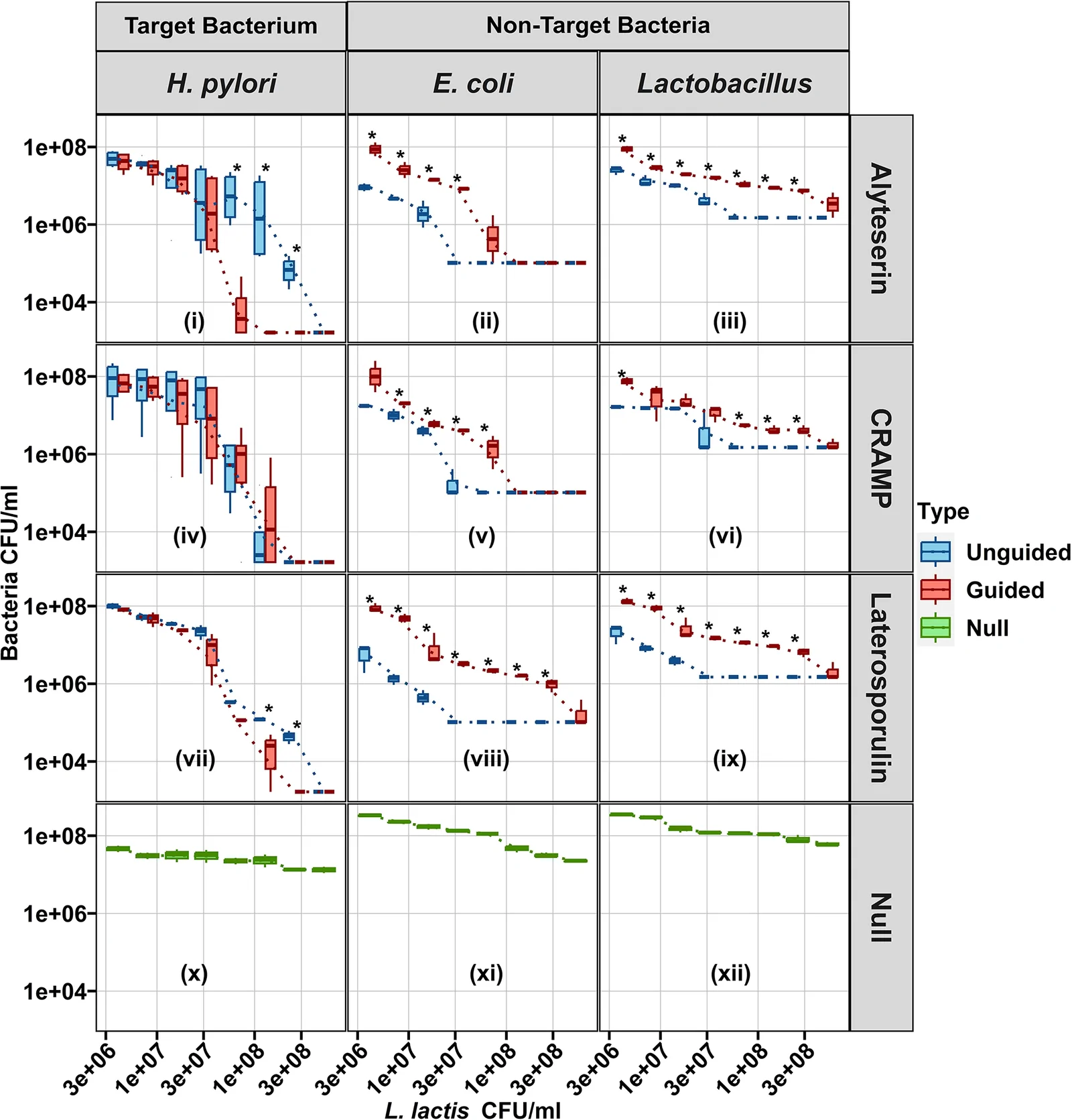
gAMP probiotics selectively kill H. *pylori* when co-cultured *in vitro*. Guided (red) or unmodified (blue) versions of three AMPs (alyteserin, CRAMP, and laterosporulin) were expressed by engineered *L. lactis,* which was co-cultured with the target (*H. pylori*) or the non-target bacteria, *E. coli* and *Lactobacillus*. Eight different initial probiotic concentrations were tested for each AMP or gAMP (x-axis), and the titer of the *H. pylori* or off-target bacterium was measured after 24 hours of co-culture (y-axis). Titers were determined starting with qPCR using *vacA* primers for *H. pylori*, DE3-T7 polymerase primers for *E. coli, recA* primers for *Lactobacillus*, and *acma* primers for *L. lactis*. The corresponding CFU values were calculated from standard curves of C_T_ versus CFU using CFU values obtained by bacterial dilution and plating (Fig. S3). The limits of qPCR detection differed between bacterial species resulting in flat-lining at different low-end levels.

*L. lactis* probiotic expressing AMPs or gAMPs controlled *H. pylori* when co-cultured *in vitro* (Fig. 3). Significantly higher toxicity was associated with gAMPs compared to AMPs for alyteserin and laterosporulin at the higher titers of probiotics (*P* < 0.02 and *P* < 0.01, respectively). However, variability in the data precluded making this conclusion across all titers. CRAMP toxicity was unaffected by the presence of a guide peptide across all probiotic titers. The presence of the probiotic itself had little impact on the growth of *H. pylori* even at high doses (Fig. 3, panels x–xii). Specifically, the empty vector probiotic (green lines) caused only a slight and insignificant decrease in *H. pylori* titer during co-culture between probiotic titers of 3 × 10^6^ and 3 × 10^8^ CFU/mL for all bacteria tested. Thus, it was the AMP component that provided a strong kill of *H. pylori in vitro*, rather than the probiotic delivery vehicle itself.

An important component of the design of our probiotic was minimizing off-target effects, such as those seen with standard antibiotics. To test the impact of gAMP probiotics on non-targeted bacteria, we chose two bacteria commonly found in the gastric microbiota. Against these off-target bacteria, the presence of the guide strongly and significantly attenuated the toxicity of all three AMPs. Against *E. coli* (Fig. 3, Column 2), all probiotic/gAMP treatments (red lines) resulted in significantly less toxicity than probiotics expressing the corresponding unguided AMPs (blue lines). The maximal toxicity differential between AMP and gAMP was 83-fold, again seen with alyteserin, at a probiotic dose of 3.2 × 10^6^ CFU/mL. Of note, all three AMPs tested have published maximal native toxicity against Gram-negative bacteria (such as *E. coli*), with much less activity against Gram-positive (such as *Lactobacillus*) (41 – 43). However, even against *Lactobacillus*, an eightfold differential toxicity between AMPs and gAMPs was seen at 6.4 × 10^6^ CFU/mL of probiotic expressing alyteserin (Fig. 3, panel iii). Overall, these data show that the gAMPs have reduced or minimal off-target effects as compared to the unguided AMPs.

### gAMP and AMP probiotics strongly control *H. pylori* in the mouse model

The therapeutic effects of probiotics expressing gAMP or AMP were examined in a mouse model of *H. pylori* infection. To maintain a consistent but natural microbiota, a single source colony of healthy mice was used throughout the experiments rather than germ-free mice. Even though other *Helicobacter* species are associated with laboratory rodents, such as *H. hepaticus or H. rodentium*, *H. pylori* does not naturally occur in mice (44, 45), so this mouse model does not address cellular infection and pathology. The model was employed to study the interaction of *H. pylori*, probiotics, gAMPs, and a complex microbiota in a natural gastric environment as a first step to evaluate the potential of gAMP probiotics for therapy or prophylactic treatment against *H. pylori*.

**Fig 4:**
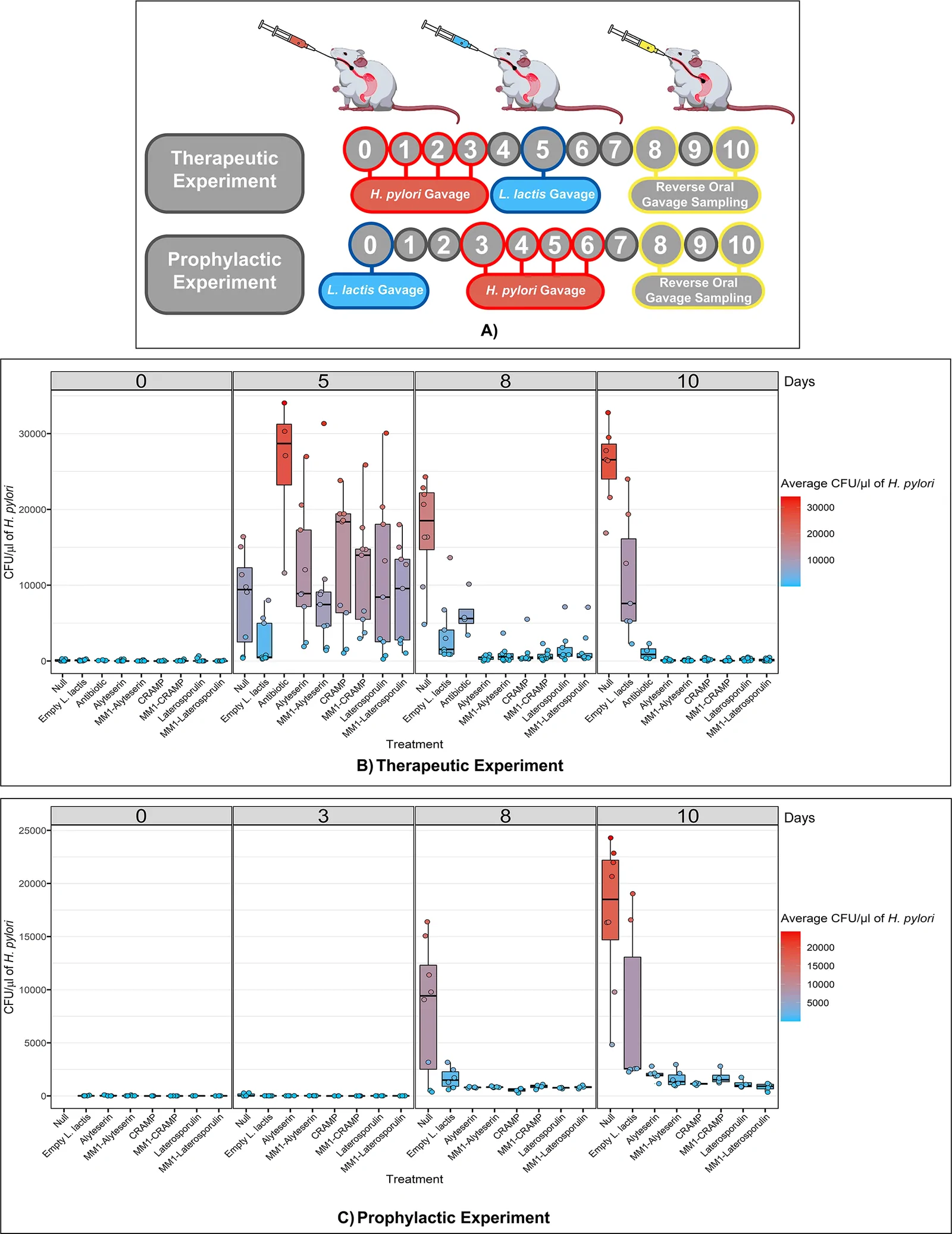
gAMP and AMP probiotics control *H. pylori* in the mouse model both as a therapeutic and a prophylactic. (**A**) For both therapeutic and prophylactic experiments, the *H. pylori* infection was established by oral gavage for four consecutive days with 250 µL of resuspended *H. pylori* (~5 × 10^7^ CFU/mL). For therapeutic experiments, the infection was followed by a dose of 250 µL of resuspended *L. lactis* (~5 × 10^7^ CFU/mL) on day 5 of the regimen. For prophylactic experiments, the probiotic was provided on day 0, followed by a *H. pylori* challenge on days 3–6. Immediately before administration of both *H. pylori* and *L. lactis*, mice stomach samples were extracted by reverse oral gavage method. Further samples were extracted on day 8 and 10 by the same method. For each treatment listed in (**B**) and (**C**), at least six mice were used. (**B**) *H. pylori* titer measured over the time course of the therapeutic experiment. On each day, probiotics expressing gAMP or AMP were compared to antibiotic treatment or negative controls. *H. pylori* titers in the reverse oral gavage stomach samples were determined by qPCR using the CFU vs C_T_ standard curve for *H. pylori* (Fig. S3). The strength of infection is color-coded. Complete data with significance values are presented in Table S2 and S3. (**C**) *H. pylori* titer measured over the time course of the prophylactic experiment. The same probiotic and negative control treatments and *H. pylori* titer determinations were used as in (**B**).

*H. pylori* gastric infection was initiated in the mice by oral gavage, followed by a single probiotic treatment at day 5 by oral gavage (Fig. 4A). To measure the *H. pylori* load change over time, mouse stomach samples collected by a novel reverse oral gavage method were analyzed by qPCR. The qPCR values were calibrated to *H. pylori* titer in a separate *in vitro* experiment (Fig. S3). Samples were taken on day 0 before introduction of *H. pylori* to record baseline qPCR readings of mouse gastric fluids. Day 5 samples were taken before the probiotic introduction to measure the extent of *H. pylori* infection prior to probiotic treatment. Days 8 and 10 samples were taken to measure the effect of the probiotic therapy on elimination of *H. pylori* infection.

Probiotic gAMP/AMP therapy provided rapid control of *H. pylori* loads in the mouse stomach (Fig. 4B). The qPCR readings quickly returned to baseline day 0 levels following treatment with the *L. lactis* probiotic expressing AMPs or gAMPs. There was no significant difference in efficacy between AMP and gAMP groups or between any of the three AMPs used. In contrast, the *H. pylori* titers of the empty vector group and the null control (no treatment) group continued to increase unabated between days 5 and 10. Specifically, AMP or gAMP therapy resulted in H. *pylori* titers 520-fold and 1,100-fold less than seen with the empty vector and null control groups, respectively. When the gAMP therapy is considered in isolation, these differences are 860-fold and 1,860-fold as compared with negative controls, while AMP therapy resulted in titers 370-fold and 800-fold less than comparison negative controls (all comparisons significant to *P* < 0.05). While antibiotic therapy resulted in a small reduction of *H. pylori*, the effect of AMP and gAMP on the reduction of *H. pylori* was significantly more effective (ANOVA, *P* < 0.05, Table S2). No significant decrease in *H. pylori* was observed in the empty vector treatment over the null control, indicating that the probiotic *L. lactis* by itself did not provide any therapeutic effect. Therefore, gAMP/AMP probiotic therapy effectively eliminated *H. pylori* infection in just 5 days in a natural mouse gastric model.

Strong protection against *H. pylori* was provided prophylactically by probiotics expressing AMPs or gAMPs. Mice were first inoculated with probiotics on day 0 followed by a challenge with *H. pylori* on day 3 (Fig. 4A). Prophylactic treatment led to lower infection levels than what was found in mice with no probiotic treatment or in mice pretreated with probiotics containing an empty vector (Fig. 4C). Specifically, the average *H. pylori* titer of AMP- and gAMP-treated mice at day 10 was 50-fold less than the infected mice with no treatment and fivefold less than the infected mice with the empty vector prophylactic control. These differences were significant (ANOVA, *P* < 0.05), as was the difference between the null and empty vector controls, pointing to a slight prophylactic effect due to the probiotic alone. No significant differences in final *H. pylori* titers were seen among the six AMP and gAMP treatments. Thus, the prophylactic use of gAMP probiotics is effective in our mouse model system.

### Probiotic gAMP/AMP treatment *in vivo* reverses the degradation of taxonomic richness caused by *H*. *pylori* infection

**Fig 5:**
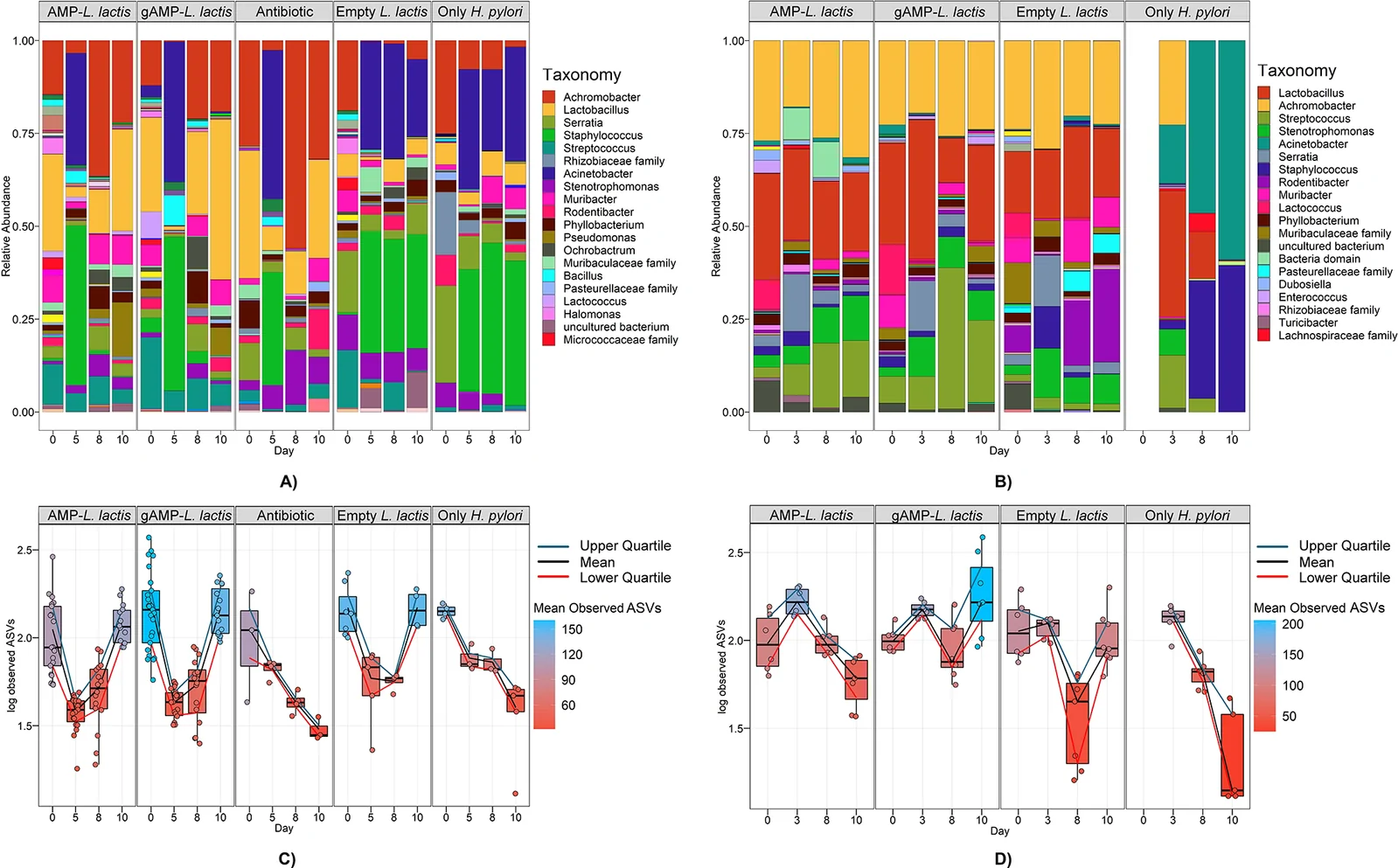
Probiotic gAMP/AMP treatment *in vivo* reverses the degradation of taxonomic richness caused by *H. pylori* infection. (**A and C**) The left two panels cover the therapeutic experiment, displaying the taxonomic analyses of the samples from Fig. 3B. (**B and D**) The right two panels cover the prophylactic experiment, with taxonomic analyses of the samples of Fig. 3C. (**A and B**) The top panels display the major genera found on each sampling day for each treatment, with *H. pylori* infection leading to domination by *Acinetobacter* and *Staphylococcus* and a relief from this domination being provided by the probiotic treatments. (**C and D**) The bottom panels chart the taxonomic richness by a simple ASV log_10_ count over time for each treatment group, with a rebound or retention of taxonomic richness in the probiotic treatment group, especially the gAMP probiotic group.

To track the population dynamics of the stomach microbiota in response to the probiotic treatments, the 350 mouse stomach samples collected for *H. pylori* qPCR analysis in both the therapeutic and prophylactic experiments were analyzed by 16S rRNA gene sequencing. For the therapeutic experiment, these results are shown in the left two panels of Fig. 5 (Fig. 5A and C). Within the first 5 days after *H. pylori* infection, taxonomic richness was greatly decreased (*P* < 0.05; Table S4). Without rescue by probiotic treatment at day 5, the mean amplicon sequence variant (ASV) count of the negative controls continued to decrease past day 5, from a high of 175 at day 0 to a low of below 20 at day 10 (all *P* < 0.05; Table S4). This decrease in taxonomic diversity was accompanied by the increased dominance of two specific genera—*Acinetobacter* and *Staphylococcus*. Despite the natural variation in initial ASV counts between mouse cohorts, significant differences in species richness were seen for the different treatment types. As expected, antibiotic treatment led to a steady decrease in the ASVs overall, to 26.7% of the day 0 mean value (*P* < 0.05; Fig. 5C; Table S4 and S5). This was in contrast to a strong rebound in species richness seen with the AMP and gAMP probiotic treatments (Fig. 5C). The highest rebound was seen with probiotics expressing gAMPs (350% increase; Table S4 and S6) and, to a lesser extent, by probiotics expressing AMP (Table S4 and S6) and by empty vector probiotics. However, ASV counts for AMP treatments were not significantly different at day 10 (*P* = 0.193) from empty vector ASV counts (Fig. 5C; Table S5) for these therapeutic experiments.

Next, we asked whether the prophylactic application of our probiotic gAMPs could prevent the loss of ASV richness accompanied by *H. pylori* infection (Fig. 5, right two panels). All probiotic prophylactic treatments preserved the taxonomic richness of the stomach after challenge with *H. pylori*, though not to the same extent. The empty vector *L. lactis* treatment group had a significant loss of ASV richness initially and did not fully recover to baseline levels (Fig. 5B and D). In contrast, the null control group (*H. pylori* only) with no probiotic pre-treatment continued to plummet in taxonomic richness on days 8 and 10 (down to 19% of the mean initial ASV; *P* < 0.01; Table S7 and S9) (Fig. 5D). As in previous experiments, the gAMP probiotic group had the most significant recovery after infection (*P* < 0.01; Table S8), and led to an ASV richness that intriguingly surpassed even the day 0 count (Fig. 5D). The AMP and probiotic empty vector treatments did not result in a significant loss or gain in species richness following the *H. pylori* challenge (Fig. 5D). The Shannon diversity and Faith phylogenetic diversity indices showed similar dynamics for both therapeutic and prophylactic experiments with AMP probiotic and gAMP probiotic helping recovery of microbial diversity post-*H. pylori* infection and protection of this diversity when used as a prophylaxis, with gAMP probiotic acting to a greater extent (Fig. S3 to S6). Overall, these data provide evidence that pre-treatment with probiotic gAMP targeting *H. pylori* can prevent the loss of microbial richness after *H. pylori* infection *in vivo*.

### Probiotic gAMP/AMP treatment protects against microbial dysbiosis

Given that the analysis of Fig. 5 revealed a central theme of taxonomic devastation caused by *H. pylori* infection, we constructed a Microbial Dysbiosis Index (MDI) to more accurately evaluate the effects of each treatment on the recovery of the gastric microbiota. We focused on taxa that are key determinants of a healthy or dysbiotic state rather than on taxonomic richness more broadly. Furthermore, an internal index of key taxa was created rather than creating an external index dependent on data from other potentially unrelated studies. To create the MDI, the 16S rRNA gene sequencing data set from Fig. 4 was used to establish a baseline “healthy” microbiota snapshot (day 0 for all mouse groups) and a dysbiotic microbiota snapshot (fifth day of *H. pylori* infection for all mouse groups) before any probiotic or antibiotic treatments had been applied.

**Fig 6:**
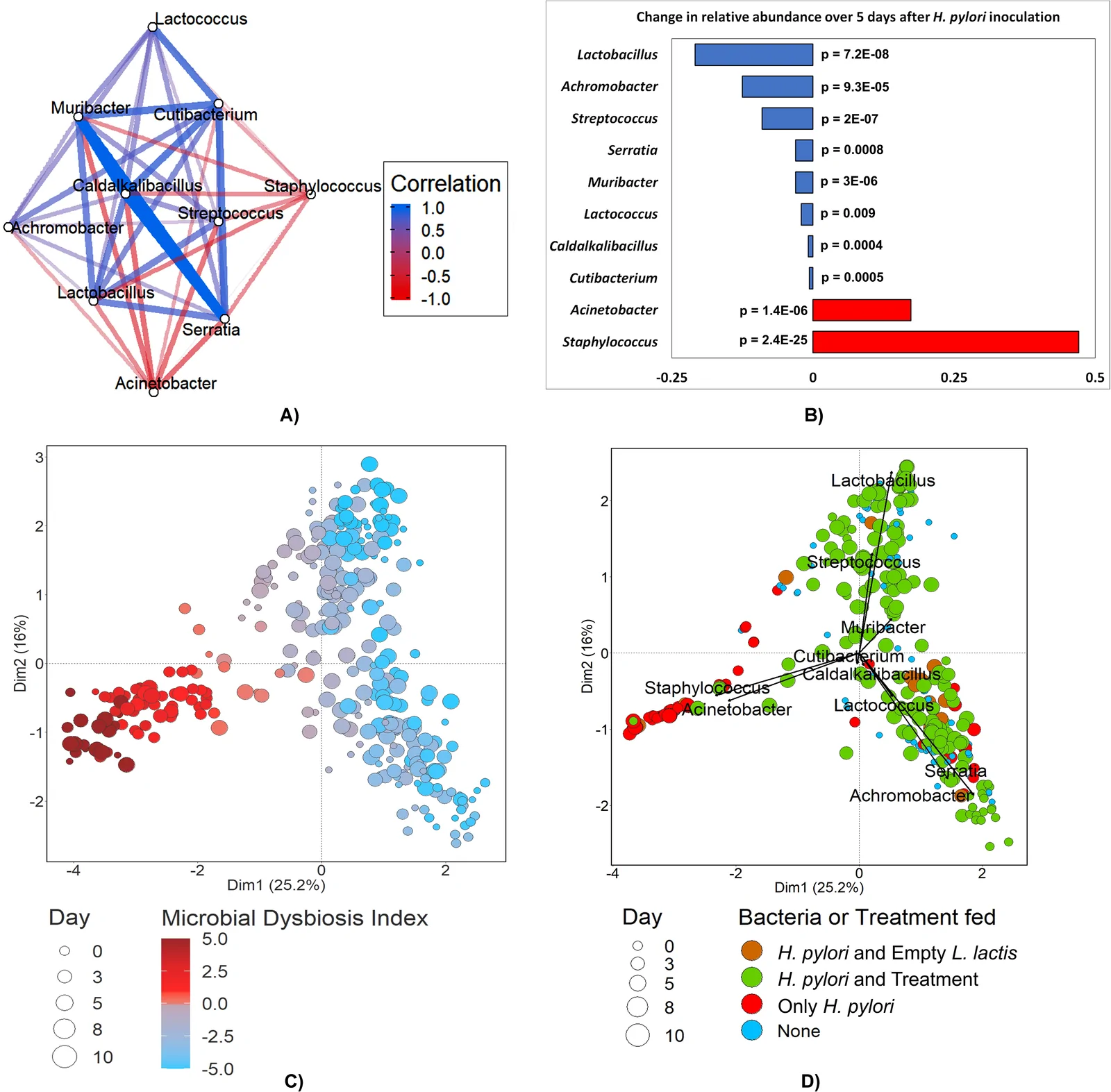
The Microbial Dysbiosis Index (MDI) comprised 10 co-varying taxa that also correlated with *H. pylori* infection. (**A**) Compositional Correlation network of 10 genera revealed by the CCREPE analysis, whose composition co-varied among the mice stomach samples on days 0 and 5, which were pre- and post*-H. pylori* infection, respectively. Eight of these were positively correlated with each other (blue lines), while two others (*Staphylococcus* and *Acinetobacter*) were positively correlated with each other but inversely correlated with the other 8 (red lines). All the correlations were significant (*P* < 0.05, *q* < 0.10). (**B**) The 10 genera that co-varied significantly also had a significant change in their mean relative abundance among the samples of day 0 vs day 5, with 8 of them decreasing following *H. pylori* infection while *Staphylococcus* and *Acinetobacter* saw a significant increase. This further validates the importance of these genera as markers for mice stomach microbial health in our experiment. (**C**) Principal Components Analysis (PCA) of the taxonomic relationship between the microbiota of all 350 samples in the study. Dysbiotic samples (red) clustered separately from non-dysbiotic samples (blue), demonstrating that MDI correlated with the taxonomic relatedness of the samples generally. Jitter was used to allow all samples to be in view. (**D**) The same PCA as in (**C**) but with ordinates overlayed corresponding to the 10 genera of the MDI, with longer vectors indicating more correspondence to the abundance of that genus. *Staphylococcus* and *Acinetobacter* are again seen as strong predictors for *H. pylori*/no treatment samples (red). No jitter was used in order to report the unaltered PCA output.


To create the MDI, we first identified taxa whose abundances varied in a similar fashion between the mouse samples collected from day 0 (pre-infected) and day 5 (infected). The CCREPE package was used to find the features (taxa) that co-varied among the samples and to determine the significance of the similarity measures for each feature pair. Every feature in the data set was analyzed against every other feature as a pair using permutation/renormalization and bootstrapping (30, 31). The top features with the most significant *P*-values (<0.05) and *q*-values (<0.10) were selected to create a correlation network illustrating the features (taxa) that were significantly associated, positively or negatively, with the *H. pylori* infection state (Fig. 5A). This analysis revealed eight genera (*Lactococcus*, *Muribacter*, *Cutibacterium*, *Caldalkaibacillus*, *Streptococcus*, *Achromobacter*, *Lactobacillus,* and *Serratia*) being positively correlated with each other and two other genera (*Staphylococcus and Acinetobacter*) being negatively correlated with the other eight, based on their relative abundance across days 0 and 5 samples (Fig. 6A). On analyzing the change in relative abundances of these 10 genera between the samples of day 0 and day 5 (Fig. 6B), all eight positively correlated genera had a higher relative abundance in day 0 (pre-infected) compared to day 5 and the other two, *Staphylococcus and Acinetobacter*, had a significantly higher abundance in day 5 (infected) compared to day 0 (Fig. 6B). This day 0 /day 5 information was then used to specify our *H. pylori* infection MDI using the general equation below. In our case, the MDI numerator would comprise *Staphylococcus* and *Acinetobacter* while the denominator would comprise the other eight genera. The abundances of these 10 genera could then be used to determine the particular MDI value for any sample of any treatment or time point.

$$MDI=\log_{10}\left(\frac{\sum \text{ Relative abundance of bacteria that increased }\text{ in abundance }}{\sum \text{ Relative abundance of bacteria }\text{ that decreased }\text{ in abundance }}\right)$$

Before using the MDI, we performed a validation step, testing a series of approaches to predict the identity of each sample as day 0 (pre-infection) or day 5 (infected). Hence, a training set was created using 30% of the data chosen randomly to train different machine learning algorithms. This was used to predict the infection status of the remaining mouse samples from the day 0 and day 5 sets. An area under curve (AUC) analysis (Fig. S6) revealed that the Random Forest (AUC = 0.913) algorithm had the best results and also that the MDI as a feature ranked better than other variables as a predictor of infection status (Fig. S7A and B).

When MDI was calculated for all 350 samples, samples clustered according to their MDI when arranged by taxonomic distance. A principal component analysis (PCA) was performed based on the taxonomic distance between each sample pair, using the top 50 most abundant genera (Fig. 6C). The PCA revealed a clear clustering of the samples with a calculated MDI over 0 [i.e., dysbiotic after *H. pylori* infection (Red)], clustering separately from the samples with a negative MDI (blue). When the abundance in each sample of each of the 10 MDI genera was overlayed as 10 ordinates on the PCA (Fig. 6D), 2 of these 10 ordinates, *Staphylococcus* and *Acinetobacter*, lay separate from the other eight ordinates. These two ordinates contained the bulk of the untreated *H. pylori* infection samples (red dots), whereas the uninfected or treated samples were generally found quite separate from these *Staphylococcus* and *Acinetobacter* ordinates. This distribution provides further validation for our MDI as a reliable tool for determining the efficacy of the probiotic treatments in protecting against dysbiosis caused by *H. pylori* infection in the mice model.

**Fig 7:**
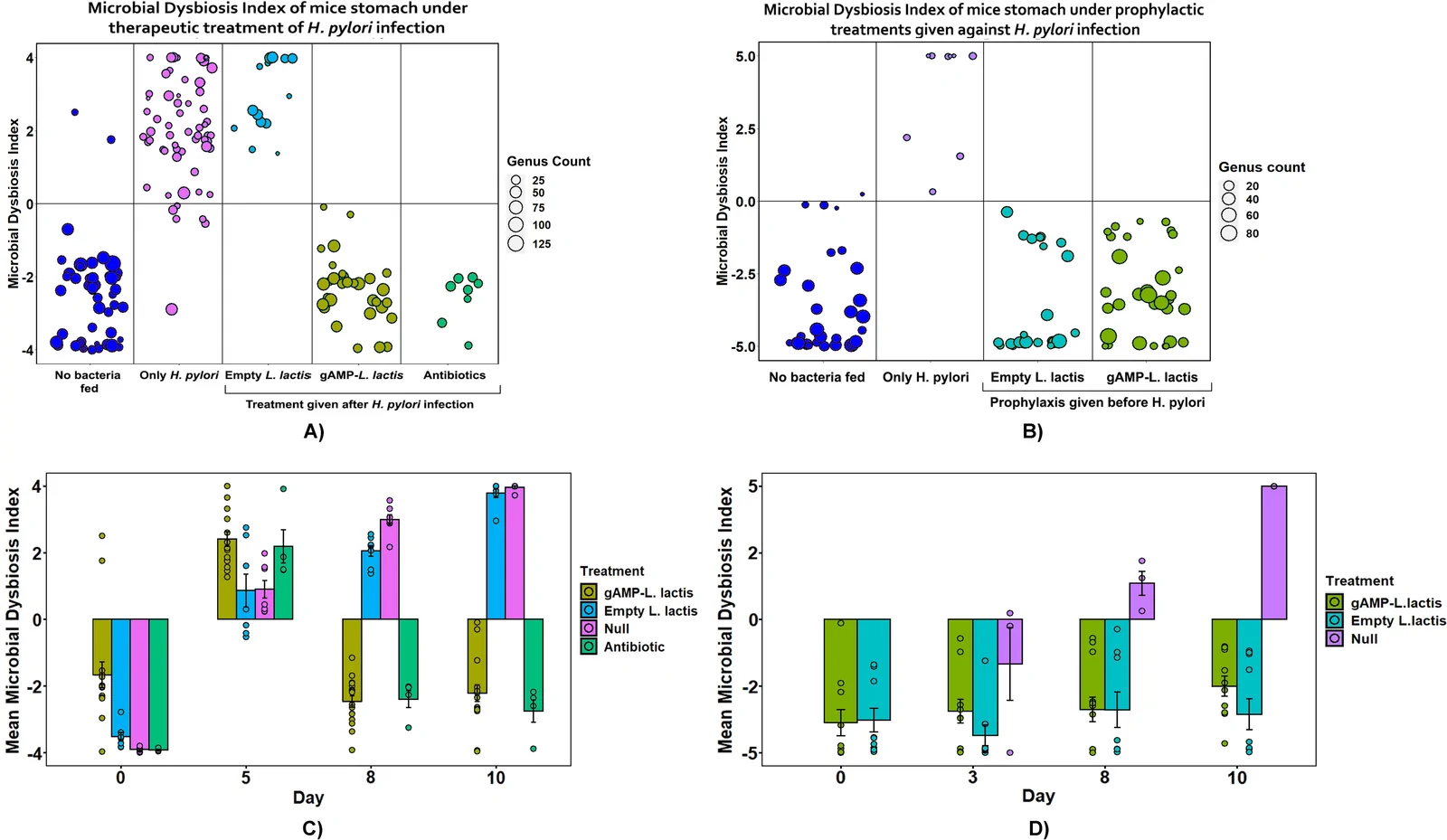
Probiotic gAMP treatment protects against microbial dysbiosis. (**A**) At the start of the therapeutic experiment (day 0), the MDIs of all samples (dark blue) were negative (healthy) and had predominantly high taxonomic richness (circle size). After *H. pylori* infection and probiotic treatment (days 8 and 10 combined), untreated (“only *H. pylori*”) and empty probiotic-treated samples were dysbiotic with low taxonomic richness, while AMP and gAMP probiotic-treated samples were healthy and had high taxonomic richness. Antibiotic treatment relieved dysbiosis but had low taxonomic richness. (**B**) In the prophylactic experiment, all probiotic treatments protected against *H. pylori*-induced dysbiosis, with gAMP probiotics promoting the most robust taxonomic richness. Infected mice without the prophylactic yielded only dysbiotic samples. (**C**) Dysbiosis in the therapeutic experiment, tracked by day. All treatments showed a negative (healthy) MDI for day 0 and a positive (dysbiotic) MDI for day 5 since these samples were collected just before oral inoculation with *H. pylori* or probiotic, respectively. The bar colors correspond to the colors and treatments in (**A**). Within 3 days, dysbiosis had been alleviated by gAMP and AMP probiotics and antibiotics but not by the empty vector probiotic. (**D**) Dysbiosis in the prophylactic experiment, tracked by day. All samples had healthy MDI values before (day 0) and 3 days after probiotic treatment, but *H. pylori* induced dysbiosis by days 8 and 10 in samples lacking prophylaxis (null treatment, magenta). All probiotic treatments protected against *H. pylori*-induced dysbiosis.

Probiotics expressing AMP or gAMP had lower MDIs and greater taxonomic richness than other treatments. Within the therapeutic group (Fig. 7A), *H. pylori-*infected mice without therapy had the most dysbiosis, followed by the probiotic/empty vector mice. Negative MDI scores were associated with greater taxonomic richness (larger diameter circles) across all groups (Fig. 7A and B). Echoing Fig. 4C, antibiotic-treated samples (Fig. 7A, rightmost box) had lower taxonomic richness than gAMP or AMP/probiotic samples. The antibiotics lowered the MDI, but perhaps this was fortuitous since the MDI calculations comprised only 10 species. Within the prophylactic groups (Fig. 7B), the MDI scores were negative (no dysbiosis) for every sample belonging to mice that were pre-treated with any type of probiotic, with only the *H. pylori*/no treatment samples having positive MDI scores, indicating that probiotics had a protective effect against dysbiosis induced by *H. pylori* infection. As in Fig. 4D, gAMP probiotic samples had greater taxonomic diversity (larger circles in Fig. 7B) than samples from the other probiotic treatments.

To determine if these treatment effects on microbial dysbiosis were changing over time, we examined the MDI for each day by treatment group. In the therapeutic experiment group (Fig. 7C), the MDI scores were negative for all treatments on day 0, but all became positive on day 5, after four consecutive days of administering *H. pylori*. On days 8 and 10, among those groups treated with the AMP probiotic, gAMP probiotic, or antibiotic treated groups, the scores returned to negative/non-dysbiotic, correlating with *H. pylori* control, while those of the probiotic empty vector and untreated control groups remained positive/dysbiotic. In the prophylactic experiment (Fig. 7D), all the prophylactic treatment groups, including the empty vector control, had non-dysbiotic MDI scores at day 0 and remained non-dysbiotic after *H. pylori* challenge (days 3, 8, and 10). The null control group for the prophylactic experiment had non-dysbiotic MDI scores on day 3, which became dysbiotic on day 8 and remained dysbiotic on day 10 after administering *H. pylori* for four consecutive days. Overall, these data show that AMP and gAMP probiotics protected the gastric microbiota from *H. pylori*-induced dysbiosis in addition to increasing species richness for both therapeutic and prophylactic applications.

## DISCUSSION

There were several findings in this study that show promise towards the development of an effective selective treatment against *H. pylori* infection. First, the presence of the MM1 guide resulted in a strong attenuation of activity against off-target bacteria *in vitro*. For example, probiotics delivering guided alyteserin AMP were 83-fold less toxic than those delivering unmodified alyteserin AMP against the off-target bacterium, *E. coli*. Even with Gram (+) *L. plantarum*, which already has a low baseline sensitivity to the AMPs used in the study, an eightfold reduction of toxicity was observed with gAMP over AMP. This selective toxicity sets these probiotic gAMPs apart from standard antibiotics, which flatten the population structure of the microbiota (21, 23) and enable pathogenic bacteria to invade and propagate (24). In addition to this selectivity, the probiotic gAMPs and AMPs exceeded the antibiotic standard in efficacy against the target bacterium, *H. pylori*.

Second, it is significant that our *in vitro* results were all achieved using probiotic co-culture to deliver the gAMP rather than purified guided AMP peptides. There are only a limited number of studies on guided AMPs and these have all relied on synthesized or heterologously expressed purified peptides. In these studies, biofilm-signaling pheromone peptides have been used as guide peptide sources (46, 47, 48) and a guide peptide was developed by biopanning against target bacterial cells (49, 50). In addition, an R-type bacteriocin naturally selective for *Clostridium difficile* strains has been engineered by receptor binding site exchange to shift selectivity to other *C. difficile* strains (51). Our probiotic gAMPs achieved a differential in toxicity between AMP and gAMP that was equal to or exceeding those reported for these purified or synthesized peptides.

Third, these engineered probiotics proved highly effective at eliminating *H. pylori* in a natural gastric mouse model. In the therapeutic experiment, the effective elimination of *H. pylori* was achieved in 5 days with a single probiotic dose. This single dose of probiotic was more effective against *H. pylori* than a single dose of antibiotic. Among the control treatments, probiotic *L. lactis* carrying an empty vector provided no therapeutic effect against *H. pylori*, indicating that gAMP/AMP was the effective agent. The prophylactic trials were also effective, showing a 20-fold less increase in *H. pylori* compared to the empty vector control. In other studies, the *E. coli* Nissle probiotic strain has been used successfully to deliver an AMP to control *Salmonella* infection in turkeys (52) and *Enterococcus* infection in mice (53). Similar to our own study, *L. lactis* expressing an unguided AMP has been used to treat *H. pylori* infection in mice (43, 54). In contrast to our study, repeated probiotic administrations (10^10^ CFU per dose) were used over a period of a month, and this resulted in only a reduction (55) rather than the elimination of H. pylori seen in our study, which needed only a single dose of engineered probiotic comprising only 10^7^ CFU. Perhaps the increased control seen in our study was due to our use of the acid-inducible P1 promoter rather than the nisin-inducible system utilized (43, 54).

Given the superior selectivity of the gAMP probiotics *in vitro* compared to the AMP probiotics, it was expected that the species richness of gAMP probiotic-treated mice would be far greater than AMP probiotic-treated mice. However, though gAMPs preserved species richness significantly better than AMPs in several instances, species richness was generally found to be similar between the two treatments. To explain this lack of differential effect on diversity, we propose that the microbial dysbiosis caused by the *H. pylori* infection was so extreme that the elimination of *H. pylori* by the engineered probiotics and the consequent waning of dysbiosis led to a strong rebound across the range of genera found in the healthy microbiota, regardless of any deleterious effects of the unguided AMPs. In other words, the magnitude of the difference in toxicity between gAMP and AMP was less than the magnitude of toxicity due to *H. pylori* and the dysbiotic microbiota that *H. pylori* infection generates.

To examine this microbial dysbiosis more carefully, we constructed an MDI. An MDI is usually generated by identifying marquee taxa that shift with a disease condition and condensing the changes in abundance values into a metric that helps measure the extent of the disease condition using microbial composition data (56). Such a shift in microbial composition or “dysbiosis” can be measured by using indicator species common to pathogen infected versus healthy microbiota found across many studies (56). Alternatively, dysbiosis can be measured using indicator species unique to the infected or healthy microbiota of a particular experimental system (57, 58). Given the great variability between the microbiota measured across published studies and the lack of investigations providing data for building a consensus microbial shift following infection of *H. pylori* in mice, we chose the second path. From our data collected from infected and healthy mouse stomachs, we identified 10 key indicator bacteria genera that correlated strongly with either the infected (day 5 samples after *H. pylori* administration) or healthy states (day 0 samples) (Fig. 5A and B) to construct the MDI. In the *H. pylori*-infected mice, two bacteria, *Staphylococcus* and *Acinetobacter*, predominated, with a drastic decrease or elimination of all other species. Like *H. pylori*, these genera have been shown to cause gastritis and hypergastrinemia, and their co-existence with *H. pylori* has been suggested to be due to their similar nature in sculpting the gastric environment, such as increasing pH, creating inflammation, and vacuolation of gastric tissue to enhance their survivability (59, 60). In these same studies, these two genera were found in patients with *H. pylori*-induced hypochlorhydria, dyspepsia, and gastritis. Thus, the rise of these two genera might be seen as a consequence of *H. pylori* colonization in the mice stomachs. The other eight microbiota genera among the ten key indicator taxa included *Lactobacillus, Streptococcus, Muribacter, Cutibacterium,* and other genera often associated in the literature with healthy mice gastric and gut microbiomes, which are known to maintain mouse gastric pH, lactate levels, and metabolite homeostasis (61 – 64). The results from the MDI analysis demonstrated that both the gAMP and AMP treatments provided the most complete rebound toward a healthy status for the microbiota from the *H. pylori*-induced dysbiotic state (Fig. 7). When considering the cohorts that were fed antibiotic and empty *L. lactis*, the MDI trend may look different from the quantitative diversity outcome seen in the raw ASV count from stomach samples because of the nature of the MDI equation. Since there were only two key marker genera that increased in abundance during dysbiosis, the change in their abundance had an exaggerated influence on the MDI score against the other eight genera in the denominator of the equation. On the whole, though, the MDI analysis of this study provides a glimpse of a severe microbial dysbiosis induced by *H. pylori* in which bacteria associated with poor health outcomes dominate in addition to *H. pylori*. The relief of this pervasive dysbiosis by the engineered probiotic may overshadow any benefits provided by the guide peptide approach.

Also to be noted are the beneficial effects of probiotic treatment towards preserving species diversity in the prophylactic treatment even without the expression of AMP/gAMP (Fig. 7B). The use of probiotics is sometimes shown to have a positive effect on species diversity following *H. pylori* infection (65, 66). Thus, in addition to the capacity of AMPs and gAMPs to eliminate *H. pylori*, another benefit of the engineered probiotic treatments may lie in the probiotic itself, not in its capacity to control *H. pylori* (Fig. 4), but to mitigate the alteration of the microbiota by *H. pylori* (Fig. 7B).

The next step in the evaluation of the utility of probiotic delivery of guided antimicrobial peptides is long-term rodent studies in the absence of *H. pylori*. In this way, the effect of gAMP versus AMP will not be masked by *H. pylori*-induced dysbiosis. If gAMPs preserve a healthy gastric microbiota long-term, the opportunity to develop a prophylactic against a gastrointestinal pathogen may present itself. Prophylactic approaches are not advisable with antibiotics due to the effects on the microbiota (23). Secondly, long term studies in the Mongolian gerbil model would allow for the study of the prevention of gastric cancer by probiotic gAMP therapy following *H. pylori* infection since this model supports the development of gastric cancer (67).

In conclusion, we have demonstrated several potential advantages of gAMP probiotics over antibiotic therapy. We have shown the high selectivity of gAMPs using an *in vitro* probiotic co-culture with *H. pylori*, sparing off-target bacteria. We have also developed a highly rapid and effective probiotic therapy for *H. pylori* in a mouse model system superior to the antibiotic control treatment. In this mouse model, a strong recovery from infection-induced dysbiosis was seen in the microbiota of the native stomach after probiotic treatment, with no recovery seen after antibiotic treatment. Though the selectivity of probiotic gAMPs *in vivo* still needs to be tested in future long-term studies, a successful mouse model has been established with this study. Finally, these engineered probiotics would be expected to be inexpensive to manufacture commercially, and the precedent of commercially available engineered probiotics appears to have been set (https://zbiotics.com/). Overall, our work, together with other emerging engineered probiotics, provides a platform to further develop precision probiotic therapy against *H. pylori* and other pathogens.

## MATERIALS AND METHODS

### Experimental model and subjects

#### Bacteria and plasmids

*Helicobacter pylori* SS1 strains (68) were acquired from Dr. James G. Fox, Division of Comparative Medicine, MIT, Cambridge, MA, and were maintained on Tryptic Soy Agar (TSA) (Thermo Scientific) with 5% sheep blood (Carolina) and Tryptic Soy Broth (Thermo Scientific) with 5% newborn calf serum (Gibco) under microaerobic conditions (<10% O_2_ and >5% CO_2_) using the EZ Campy Pouch System (BD GasPak) at 37°C. *Lactococcus lactis* MG1363 (LMBP 3019) was acquired from the Belgian Coordinated Collections of Microorganisms (BCCM) and was maintained in propagated in M17 broth and agar (Thermo Scientific) supplemented with 0.5% glucose (Thermo Scientific) and 5 µg/mL of Erythromycin (Thermo Scientific) for screening cloned bacteria at 28–30°C. Electrocompetent *L. lactis* were prepared by washing overnight cultures twice with an ice-cold 0.5 M sucrose and 10% glycerol solution. The plasmid pT1NX (LMBP 3498) was acquired from BCCM, cloned into *L. lactis* through electroporation (2,000 V, 5 ms), and outgrown in M17 medium. *Lactobacillus planatarum* was acquired from ATCC (NCIB 8014) and grown on MRS broth/agar (Thermo Scientific) at 37°C and 55 CO_2_ conditions. *E. coli* 10β (DH10B derivative) was purchased from New England Biolabs (NEB #C3019I) and was grown in Luira-Betani (LB) broth/agar (Thermo Scientific) at 37°C with aeration. The same strain was used for both cloning and as a target bacteria in *in vitro* experimentation.

#### Mice

C57BL/6 mice (6–8 weeks old) were purchased from Jackson Laboratories and kept in groups of two females and one male for breeding and propagation. All mice were housed in the Baylor Sciences Building (BSB) Vivarium, allowed free access to water and diet, and provided with a 12-hour light/dark cycle. All the experiments were performed under Biosafety Level 2 conditions in the BSB Vivarium (IACUC Reference #: 1240636) under the supervision of Dr. Ryan Stoffel, Attending Veterinarian and Animal Program Director. Neonates were weaned after 14 days and were only used for experiments after 6 to 8 weeks of maturity and/or reaching 25 g of body weight. Each experiment cohort had at least six mice for each treatment regimen, with equal number of males and females. Animals were stored in groups of three; for males that were not part of the same litter, they were kept solitary. The cage bedding material was changed every 10 days. For animals used in experiments and infected with bacteria, they were sacrificed immediately afterward by the CO_2_ asphyxiation method.

### Methods

#### Generation of the VacA-4S *H. pylori* VacA knockout mutant

To create the VacA-4S *H. pylori* VacA knockout mutant, stop codons replaced amino acid codons at four positions to ensure translational knockdown. As a prelude to mutagenesis, donor DNAs were synthesized from the *H. pylori* 60190 nucleotide sequence (accession number U05676) in two overlapping parts. Part A covers the 173 bp of 3′-end of *cysS*, 229 bp intergenic region, and 5 bp of 5′-end of *vacA* ORF. The nucleotide sequence of part A was altered to GGAT to create a BamHI within the intergenic region (29 bp downstream from *cysS*) for subsequent incorporation of chloramphenicol acetyltransferase (*cat*) cassette as a selection marker. Part A was then cloned in pUC57 plasmid and further modified by ligating a *cat* gene in the newly-created BamHI site. Part B DNA was synthesized and contained 100 bp of intergenic region and 500 bp of the 5′-end of *vacA* ORF. In the synthesized Part B DNA, stop codons occurred at amino acid positions 4, 5, 23, and 27, substituting glutamine, glutamine, leucine, and threonine, respectively. Parts A and B were amplified using their respective primer pairs, VacA-A (forward: TAGGCGTGAGTGAAAGCGAAAAACAAGA; reverse: TCCATTTCTTTCCTTTCTTCTTTTC) and VacA-B (forward: TATTTATAGCCTTAATCGTAAATGCAACAG; reverse: CAAGCGCAAGGTGGCTTTTTGCATATT). These pieces were then joined to each other using splice-by-overlap PCR using primer pairs VacA-A forward/VacA-B reverse with Q5 DNA polymerase (New England Biolabs). *H. pylori* 60190 wild-type strain was naturally transformed using the spliced PCR products to produce the isogenic 4xStop *H. pylori* VacA knockout mutant. Transformants were selected based on chloramphenicol resistance on GC agar plates and further screened by colony PCR to confirm the desired DNA part was incorporated. Finally, all stop codon insertions were confirmed by nucleotide sequencing.

#### Flow cytometry

Flow cytometry was used to measure cellular binding of the MM1-GFP conjugate protein against the targeted *H. pylori* strains 60190, 60190:v1, and 60190vacA-4S as well as against the off-target bacteria, *L. plantarum* (NCIB 8014), *Escherichia coli* K-12, *Pseudomonas aeruginosa* (ATCC 27853), *Staphylococcus aureus* (SA113/ATCC 35556), and *Alcaligenes faecalis* (ATCC 8750). *H. pylori* and *L. plantarum* strains were grown for 48 hours in TSB and MRS media under microaerophilic conditions (<10% O_2_ and >5% CO_2_) using the EZ Campy Pouch System (BD GasPak) at 37°C. *E. coli* and *P. aeruginosa* were grown overnight in LB media at 37°C. *S. aureus* and *A. faecalis* were grown overnight in BHI media at 37°C. Strains were standardized to an OD_600_ of 1.0 and diluted 1:20 in 1× PBS supplemented with 125 µg/mL unguided GFP or MM1-GFP and incubated for 30 minutes at 37°C, shaking at 180 rpm. Cells were washed and resuspended in 1× PBS and flowed through a BD FACSVerse system (BD Biosciences, Franklin Lakes, NJ, USA). Cells were excited with a blue 488 nm laser using a 488/10 bandpass filter. For each sample, fluorescence intensity measures were recorded for a total of ≥4,000 events collected in triplicate. Data were analyzed using FCS Express (De Novo Software, Pasadena, CA, USA).

#### Confocal microscopy

Confocal microscopy was used to visualize the cellular binding of the MM1-GFP conjugate protein against the target and off-target strains grown under the culture conditions used for flow cytometry. Strains were standardized to an OD_600_ of 1.0 and diluted 1:50 in 1× PBS supplemented with 200 µg/mL unguided GFP or MM1-GFP and incubated for 60 minutes at 37°C, shaking at 180 rpm. After initial incubation, the cells were stained by the addition of 4× Cellbrite 640 membrane stain and further incubated for 30 minutes. Cells were then washed with 1× PBS and fixed with 4% formaldehyde in PBS for 20 minutes at 4°C. After fixing, cells were washed in 1× PBS, added to a chambered coverglass (Nunc), and stored at 4°C. Microscopy was carried out using an Olympus FV-3000 confocal microscope, excited with a 488 nm and 640 nm laser to visualize GFP and the membrane stain, respectively.

#### Cloning antimicrobial peptides and guided antimicrobial peptides in *L. lactis*

The ORFs of the AMPs (Table S1), codon-optimized for *Lactococcus lactis*, were cloned into the pTKR plasmid (Fig. S1), an *L. lactis*/*E. coli* shuttle vector developed in the Kearney Lab from the *L. lactis* plasmid, pT1NX (38) by adding an *E. coli* ori site and kanR gene for propagation and selection of *E. coli* clones. This plasmid includes the P1 acid-inducible promoter and the usp45 signal peptide for transport out of the cell. The ORFs were amplified from a gBlock (IDT) by PCR (1 minute melting at 95°C; 35 cycles of 15 seconds melting at 95°C, 15 seconds annealing at T_m_ + 3°C, 30 seconds extension at 72°C; and 5 minutes elongation at 72°C) using respective primers and pasted into the pTKR plasmid using restriction enzyme cutsites post agarose gel purification. The recombinant plasmid was electroporated into *E. coli* 10β cells and plated out on LB agar (Thermo Scientific) plate with kanamycin (MP Biomedicals) to pick successfully cloned colonies. Post-colony-PCR screening, cloned colonies were picked and propagated in LB liquid media with kanamycin (25 µg/mL; Thermo Scientific). The pTKR plamids with AMPs cloned were extracted from the pelleted liquid culture using the Plasmid Extraction Kit (Promega) and electroporated into *L. lactis* MG1363 (LMBP 3019) cells, followed by erythromycin selection on GM17 plates.

#### *In vitro* assay: co-culture of engineered probiotic *L. lactis* to measure the control of *H. pylori*

*L. lactis* clones engineered to express AMP or gAMP were propagated from glycerol stocks and grown in GM17 broth overnight with erythromycin (5 µg/mL) with no shaking. *H. pylori* SS1 stocks were first propagated on blood-TSA overnight under microaerobic conditions (<10% O_2_ and >5% CO_2_). Colonies from these plates were then transferred to TS broth with 5% newborn calf serum and grown overnight under microaerobic conditions. The *L. lactis* cultures were serially diluted in a 96-well culture plate with TS broth to make up a volume of 100 µL. To each well, 10 µL of the overnight *H. pylori* culture were added, and each well volume was brought up to 200 µL with more TS broth. The plate was left to grow overnight in microaerobic conditions. After 24 hours, the well contents were transferred to a 96-well PCR plate. That PCR plate was sealed, heated for 15 minutes at 100°C, and then chilled at 4°C for 5 minutes. This plate was then centrifuged at 2,000 *g* for 2 minutes, and the supernatant was used as the template for qPCR.

qPCR was carried out using primers for the VacA gene to quantify *H. pylori* titer (forward: 5′-ATGGAAATACAACAAACACAC-3′; reverse: 5′-CTGCTTGAATGCGCCAAAC-3′) and primers flanking the *L. lactis acma* gene were used to quantify *L. lactis* titer (forward: 5′ GGAGCTCGTGAAAGCTGACT 3′; reverse: 5′ GCCGGAACATTGACAACCAC 3′). The qPCR used SYBR Green as the amplification dye and ROX as the passive dye, and the thermal cycler had 2 minutes melting at 95°C followed by 40 cycles of 15 seconds melting at 95°C, and 1 minute of annealing/extension at 60°C, ending with a melt curve. Standard curves for *H. pylori* and *L. lactis* were constructed by determining C_T_ values from the qPCR data for different dilutions of the overnight cultures of the respective bacteria (1/10, 1/100, 1/10^3^, and 1/10^4^) in the qPCR plates, and the CFUs for the dilutions were determined by plating on their respective agar plates. The same procedure was followed with the off-target bacteria where *Lactobacillus plantarum* and *E. coli* were co-cultured with serially diluted cultures of *L. lactis* for 24 hours, and the titers of the off-target bacteria were determined by qPCR using primers for species-specific genes for either bacterium (DE3-T7 polymerase for *E. coli* and *recA* for *L. plantarum*). The amount of *L. lactis* added to the co-cultures of all the three assays ranged from approximately 4 × 10^6^ to 5.12 × 10^6^ CFU/mL. The statistical difference between any data sets was performed by ANOVA. All the species specific primers are present in Table S10.

#### Administering *L. lactis* and *H. pylori* to mice by oral gavage and sample collection

All animal studies were approved by the Baylor University Institutional Animal Care and Use Committee. C57BL/6 mice were bred in-house and maintained in a specific-pathogen-free facility in adherence with the NIH Guide for the Care and Use of Laboratory Animals. Cultures of probiotic and *H. pylori* were grown out and fed to mice by oral gavage. Briefly, the *L. lactis* cultures were propagated overnight in GM17 broth with erythromycin (5 µg/mL) and no shaking. The overnight cultures were centrifuged at 4,000 *g* for 15 minutes at 4°C. The pellets were resuspended in sterile PBS. *H. pylori* SS1 stocks were grown overnight on blood-TS agar under microaerobic conditions and then scraped by a sterile loop and resuspended in sterile PBS. Both bacterial suspensions were fed to the mice using 1.5 oral gavage needles not exceeding half their stomach volume (~250 µL). The CFU of the resuspension being fed was determined by diluting the resuspension 1/10^3^ and 1/10^4^ times and plating on appropriate plates. For both *L. lactis* and *H. pylori*, the inoculum sizes were kept to ~5 × 10^7^ CFU/mL. Samples were taken using a reverse oral gavage method invented for this study. Pre- and post-inoculation samples from the mouse stomach were collected by flushing the mouse stomach with excess PBS (~250 µL) using a gavage needle, flushing up and down twice without drawing any substantial volume of fluid out. The plunger was then used to draw out 50–75 µL of flushed stomach fluid.

Four different schemes of bacterial inoculation were designed to cover each of the experimental types: probiotic therapy, antibiotic therapy, probiotic prophylactic, and the null (no treatment) control.

For the probiotic therapy, stomach samples were collected on day 0 before *H. pylori* inoculation. Over the next three days, resuspended *H. pylori* were fed by oral gavage once daily. On day 5, stomach samples were collected to test for *H. pylori* presence, followed immediately by the probiotic therapy, which consisted of a single oral gavage feeding of resuspended *L. lactis* carrying either AMP, gAMP, or the control empty pTKR vector. Follow-up stomach samples were collected on days 8 and 10.

The antibiotic therapy was performed identically to the probiotic therapy, with the substitution of an antibiotic cocktail (amoxycillin:tetracycline :: 4.5:4.5 mg/25 g of mice) fed to the mice by oral gavage in place of the probiotic on day 5.

For the probiotic prophylactic, stomach samples were collected on day 0, followed immediately by *L. lactis* inoculation carrying one of the three pTKR vectors as for the probiotic therapy. On day 3, stomach samples were taken by reverse oral gavage, followed immediately by a challenge inoculation with *H. pylori* by oral gavage, with daily *H. pylori* challenge inoculations for a total of three consecutive days. Stomach samples were collected on days 8 and 10 to test for *H. pylori* presence. For the null control mice, stomach samples were collected on day 0 before *H. pylori* inoculation, followed by daily *H. pylori* inoculations for a total of three consecutive days. Stomach samples were then collected on days 5, 8, and 10 to test for *H. pylori* presence. Six mice were used per AMP and gAMP treatment for both the probiotic therapeutic and probiotic prophylactic treatments. Six mice each were also used to constitute the null control group, the antibiotic therapy group, and the empty vector (pTKR) group for both the probiotic therapeutic and prophylactic experiments.

#### PCR assay for probiotic or *H. pylori* from mouse stomach samples

The stomach samples collected by reverse oral gavage were heated at 100°C for 15 minutes, chilled at 4°C for 5 minutes, and served as PCR templates. To verify the presence of *L. lactis*, PCR primers were used specific for the pTKR vector (forward: 5′-GCCTGAGCGAGACGAAATAC-3′, reverse: 5′-TTATGCCTCTTCCGACCATC-3′). For qPCR to quantify *H. pylori* titer, primers were used specific to the VacA gene (forward: 5′-ATGGAAATACAACAAACACAC-3′, reverse: 5′-CTGCTTGAATGCGCCAAAC-3′). Standard curves for *H. pylori* against the C_T_ values were constructed by including different dilutions of the overnight cultures of *H. pylori* (1/10, 1/100, 1/10^3^, and 1/10^4^) in the qPCR and plating those dilutions on respective plates to determine the corresponding CFU/mL values. The CFU/mL values for each sample were determined by plotting the C_T_ values against the standard curve built as described previously. The qPCR running parameters were identical. The statistical difference between any data sets were performed by ANOVA.

#### Illumina 16S rRNA sequencing of mouse stomach samples

The method was first validated by sending samples to an external service (MR DNA, Lubbock, TX) for Illumina 16S rRNA sequencing. Subsequently, all sequencing was performed in-house using an Illumina MiSeq. The stomach sample supernatants used as qPCR templates also served as templates for in-house Illumina sequencing. The sequencing was done as described in the Earth Microbiome Project (69). Briefly, the templates were amplified with 16S V4 primers (forward: 5′-TCG TCG GCA GCG TCA GAT GTG TAT AAG AGA CAG GTG YCAGCMGCCGCGGTAA-3′, reverse: 5′-GTC TCG TGG GCT CGG AGA TGT GTA TAA GAG ACA GCC GYCAATTYMTTTRAGTTT-3′) (69) and then with Illumina index primers (70) with subsequent clean-up and purification with AMPure magnetic beads (Beckman Coulter). The samples were normalized to a uniform concentration and pooled into a library with a concentration of around 4 nM. The pooled library was denatured (with 0.2 N NaOH) and further diluted to a concentration of 20 pM. The library was then spiked with PhiX phage DNA (20 pM) to make up to 20% of the resultant mixture. The sequencing was performed using the Illumina MiSeq v3 kit in an Illumina Miseq machine that generates up to 2 billion paired reads of up to 300 bases long and requires a runtime of 72 hours for 600 cycles. The reads were filtered for a q30 score of at least 85% or above.

#### 16S sequencing data analysis

The sequencing data were analyzed further downstream using QIIME2 (71, 72) with the default parameters used in QIIME2 plugins unless otherwise mentioned. The raw data were demultiplexed, denoised (dada2), and analyzed for taxonomic abundance using default QIIME2 plugins on the Kodiak High-Performance Computing (HPC) Cluster hosted by High Performance and Research Computing Services, Baylor University. The alpha diversity (Shannon, Faith Phylogenetic Diversity, and ASV count) and beta diversity (Bray-Curtis, Jaccard, Weighted and Unweighted Unifrac) analyses were performed using QIIME2 plugins, and further statistical analysis and the visualization of the data were performed on R. The resultant taxonomic abundance data (at genus level) was analyzed using the CCREPE package in R (73) with the microbial community of the mouse stomach at day 0 compared against the community from the samples taken at day 5 after three consecutive days of *H. pylori* inoculation to determine the correlation between the taxa according to relative changes in abundance upon addition of *H. pylori*. The resultant coterie of genera [abs(sim.score)>0.2, *P*-value < 0.05, *q*-value <0.10, iterations = 1,000] was then used to construct the equation for MDI equation. The correlation networking of the CCREPE output was done in R using the *ggraph*, *igraph*, and *corr* packages. Principal component analysis based on the relative abundance data was performed using the *factoextra* package. Every other data visualization used default tools in the *tidyverse* and/or *ggplot2* packages. To determine the importance of the MDI score against other variables to identify the dysbiotic nature of the samples using machine learning, we used the Random Forest Classifier available in *scikit-learn 0.23.0* on Python 3.5. For training the algorithm, we split the day 0 and day 5 samples into a 0.3:1 ratio with the abundances of the 10 bacteria generated by CCREPE, the MDI score, and the ASV count of the sample as input variables.

## Data Availability

Original, unprocessed data are available through NCBI SRA using the BioProject Accession number PRJNA885498. The paper does not report any original code. All the packages and programs used in the paper have been listed in Materials and Methods. Further information and requests for resources and reagents should be directed to and will be fulfilled by the lead contact, Christopher Kearney (chris_kearney@baylor.edu). Any additional information required to reanalyze the data reported in this paper is available from the lead contact upon request. All unique reagents generated in this study are available from the lead contact with a completed Materials Transfer Agreement.

## SUPPLEMENTAL MATERIAL

Supplemental figures and tables spectrum.02014-23-s0001.pdf Fig. S1 to S9 and Tables S1 to S10.

ASM does not own the copyrights to Supplemental Material that may be linked to, or accessed through, an article. The authors have granted ASM a non-exclusive, world-wide license to publish the Supplemental Material files. Please contact the corresponding author directly for reuse.
