# Supplementary material for: Control of Helicobacter pylori with engineered probiotics secreting selective guided antimicrobial peptides
Source: Microbiol Spectr. 2023 Sep 15;11(5):e02014-23. doi: 10.1128/spectrum.02014-23 (PMC10580918; doi:10.1128/spectrum.02014-23)
Supplement: Supplemental figures and tables — Fig. S1 to S9 and Tables S1 to S10. [file spectrum.02014-23-s0001.pdf]

Supplemental Data

Fig. S1. The vectors used to clone probiotic *L .lactis*. pTKR was created by cloning Kanamycin resistance and *E. coli* oriC site genes in the pT1NX vector.

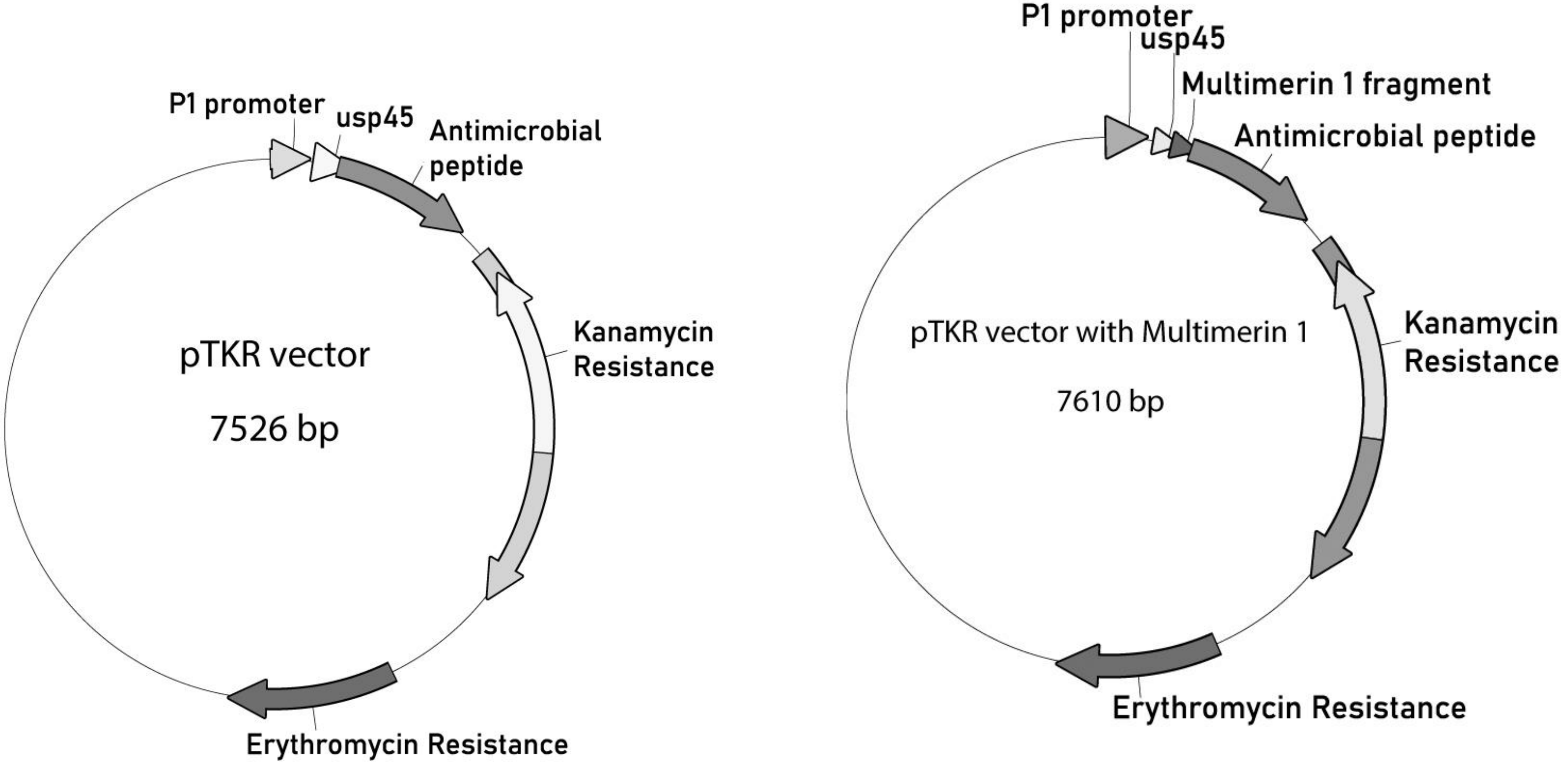

Fig. S2. **Flow cytometry of MM1-GFP off-target treated cells.** Plotted are the fluorescence intensity measures of (Panel A) *H.pylori 60190* ΔvacA , (Panel B) *P.aeruginosa* , (Panel C) *S.aureus* cells and (Panel D) *A. faecalis* . Each species was untreated (red), treated with GFP (green) or MM1-GFP (dark blue) with averaged median fluorescence from triplicate samples; Standard deviation shown; statistical significance (One-way Anova, ns- not significant, ** P ≤0.01 and *** P ≤0.001).

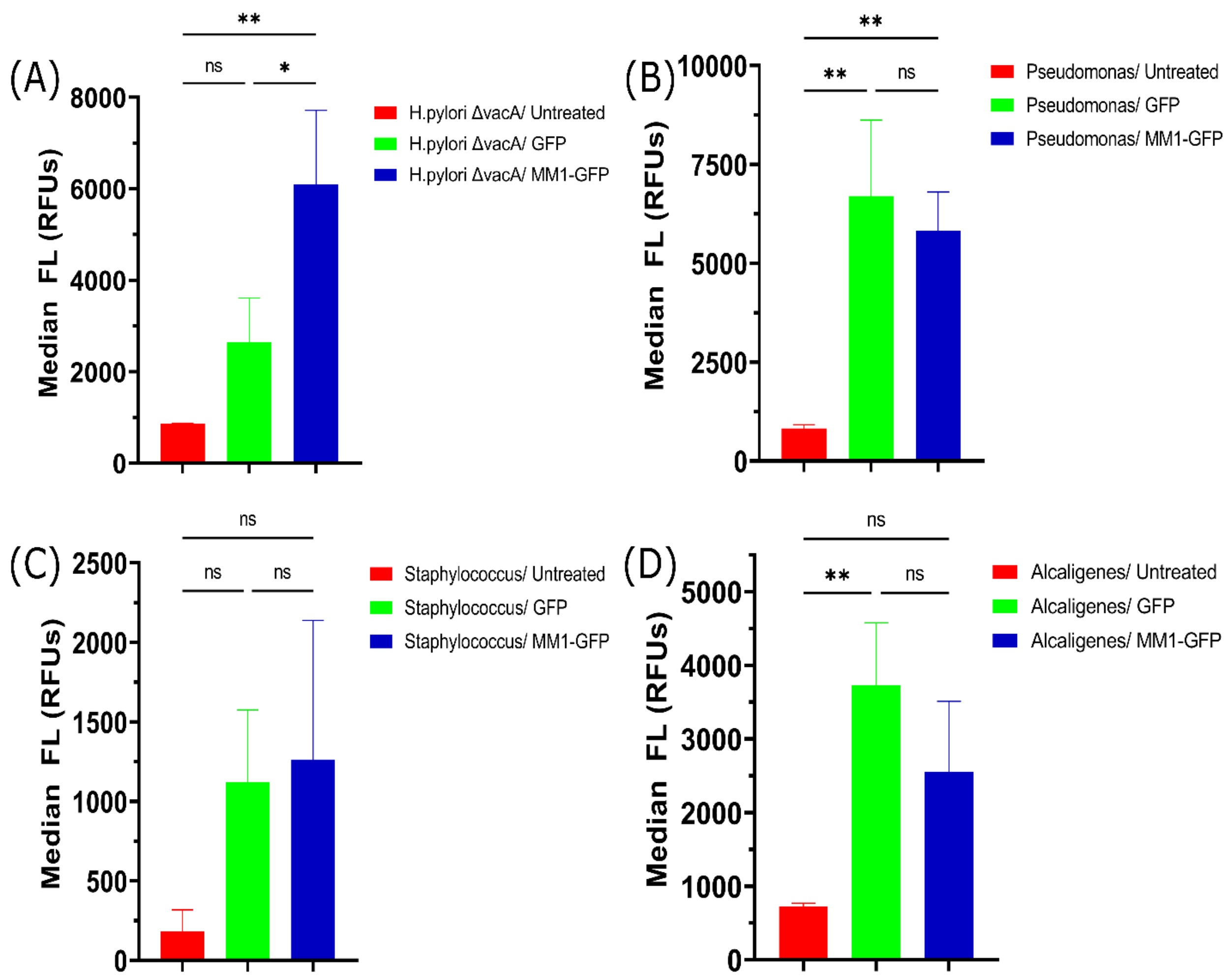

Fig. S3 *H. pylori* (a), *Lactobacillus* (b), and *E. coli* (c) standard curves plotted with $\log_{10}$CFU values obtained by plating them on respective agar plates and the C_t values obtained by qPCR of the same culture

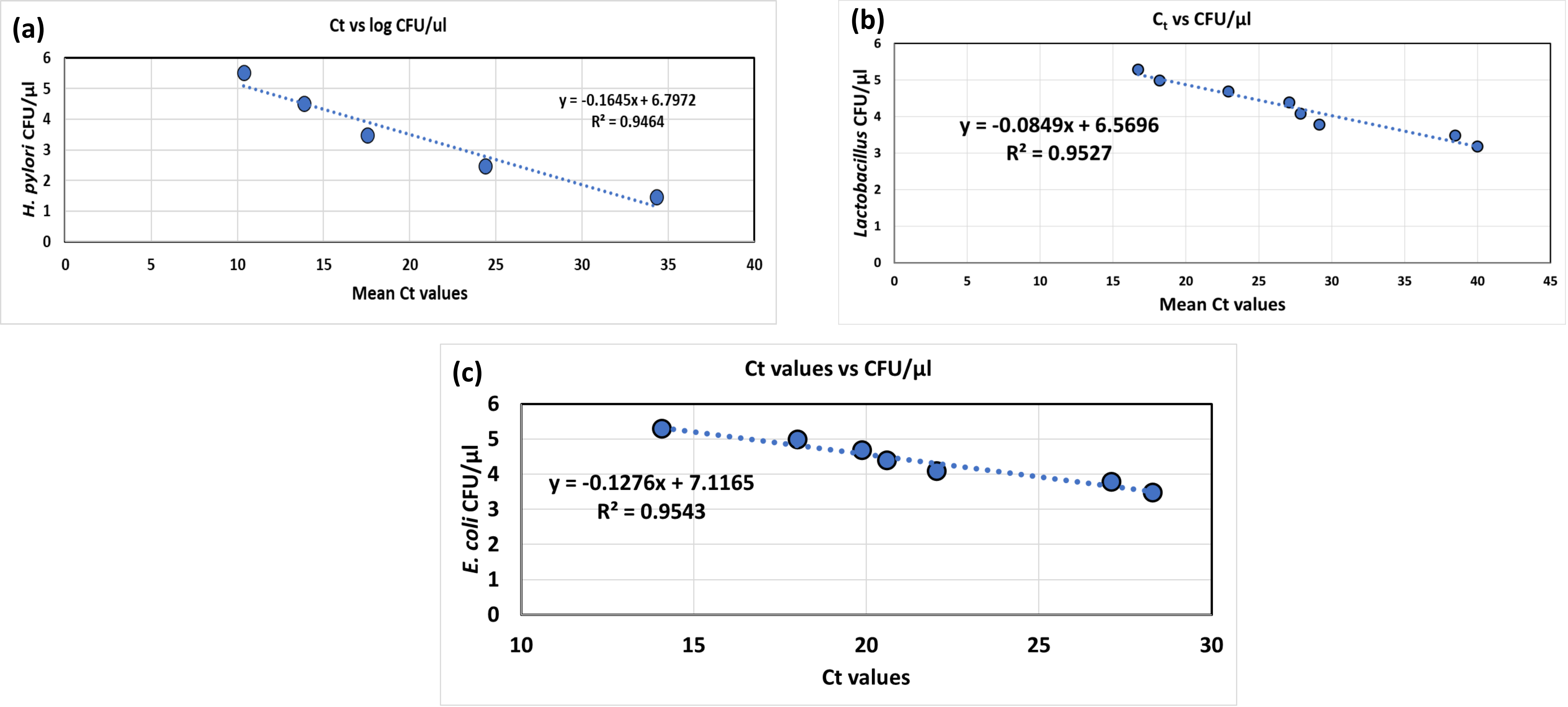

Fig. S4. Shannon diversity index for each therapeutic treatment cohorts at each day of the experiment

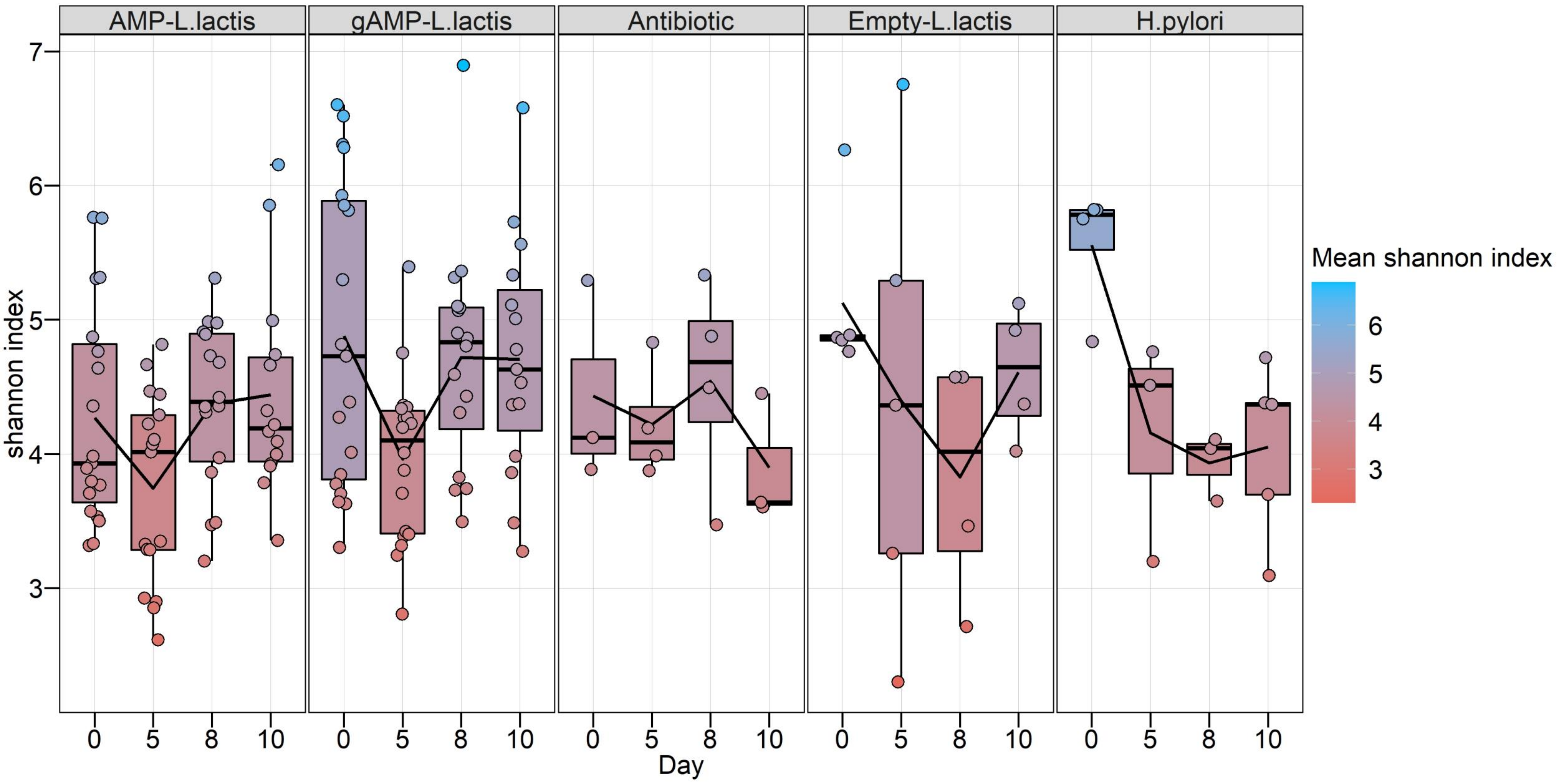

Fig. S5. Shannon diversity index for each prophylactic treatment cohorts at each day of the experiment

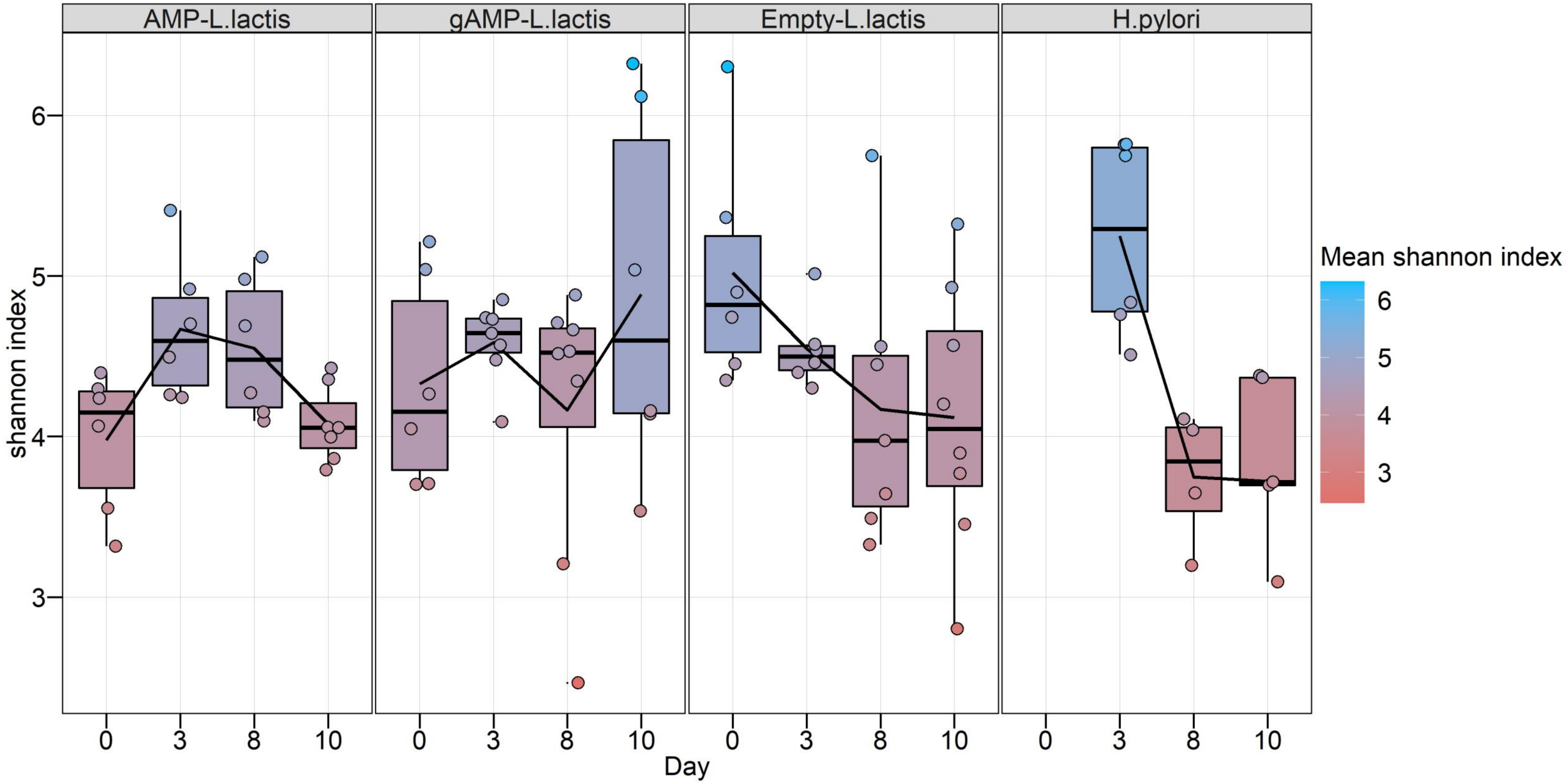

Fig. S6. Faith phylogenetic diversity index for each therapeutic treatment cohorts at each day of the experiment

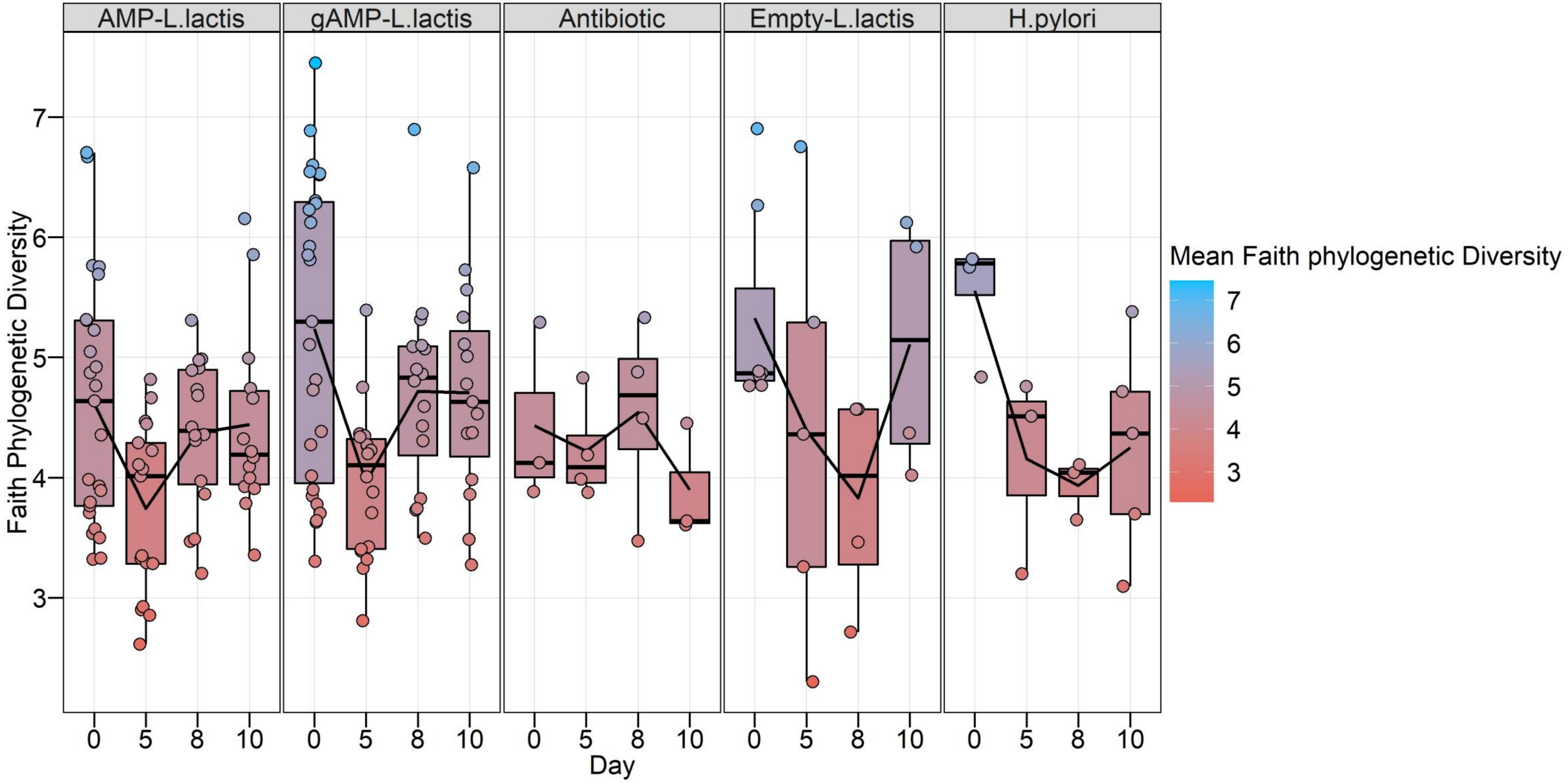

Fig. S7. Faith phylogenetic diversity index for each prophylactic treatment cohorts at each day of the experiment

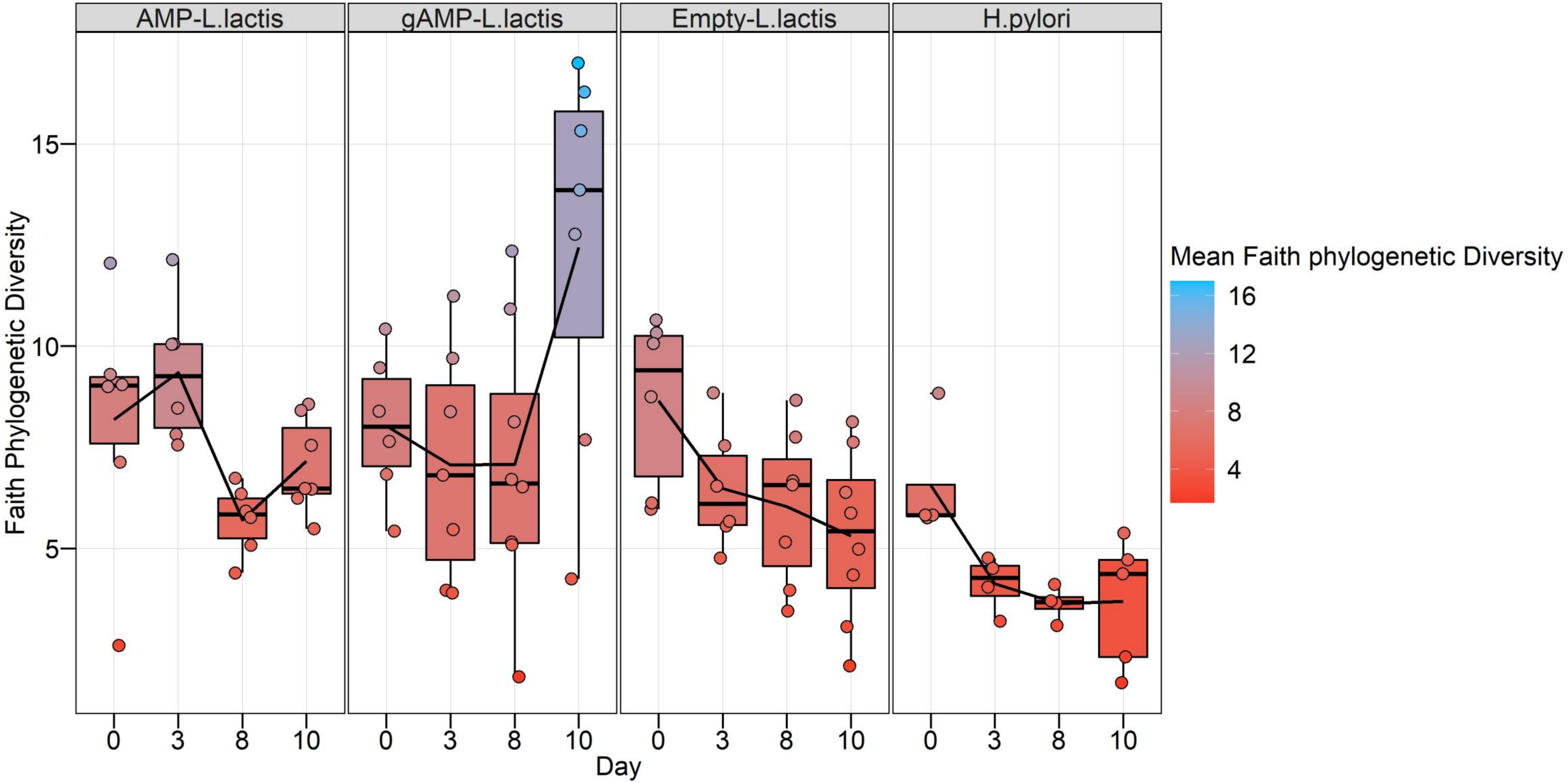

Fig. S8. AUC scores of algorithms using MDI and relative abundance of ten bacterial genera to predict the dysbiotic state of the sample i.e., fed only *H. pylori*.

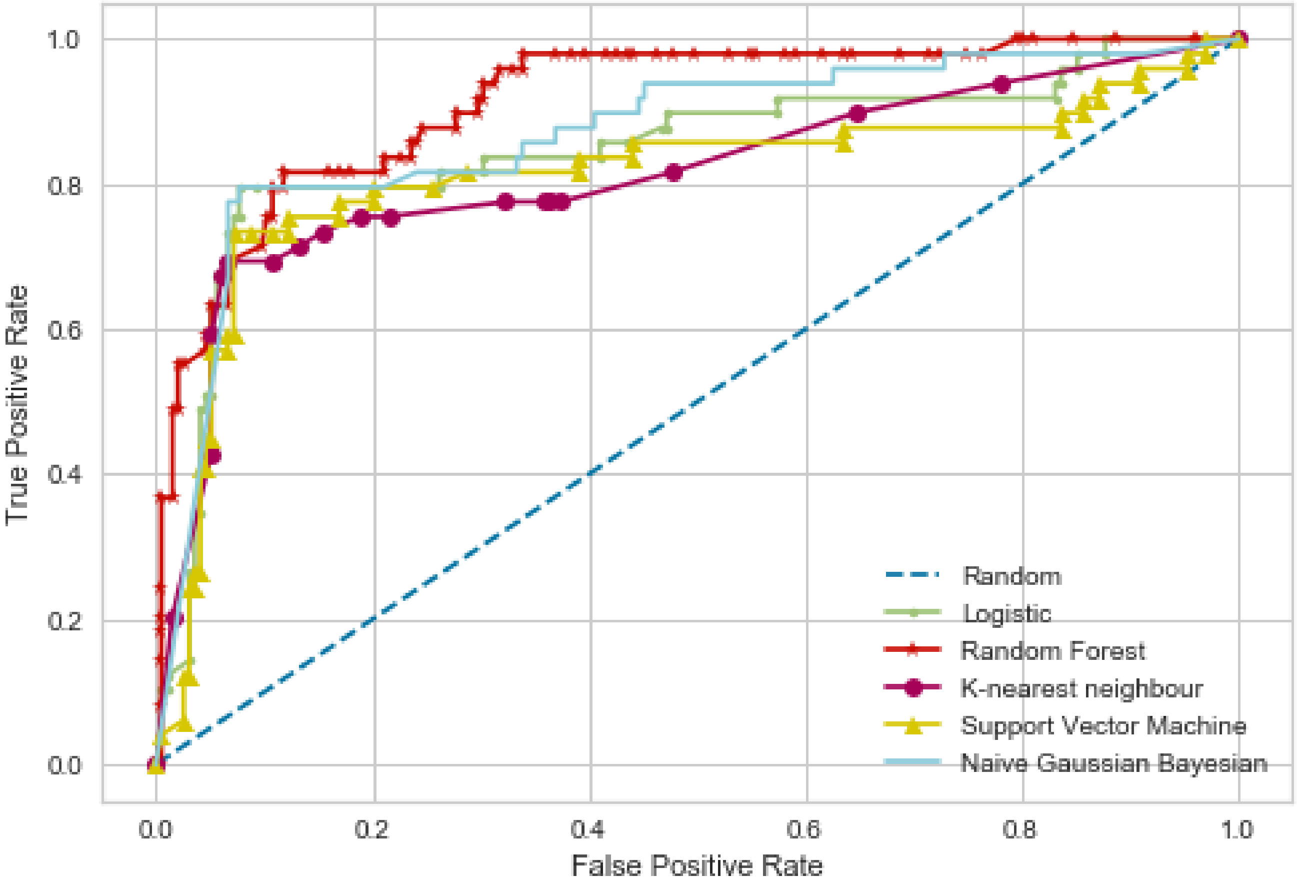

AUC Scores:
Logistic Regression: 0.8484563055991627
Random Forest Classifier: 0.9133437990580847
K-Nearest neighbor: 0.8154892726321298
Support Vector machine: 0.8122448979591834
Gaussian Naive Bayesian model: 0.8695970695970696

Fig. S9. Spearman (A) and Pearson (B) ranking of features used in the Random Forest classifier

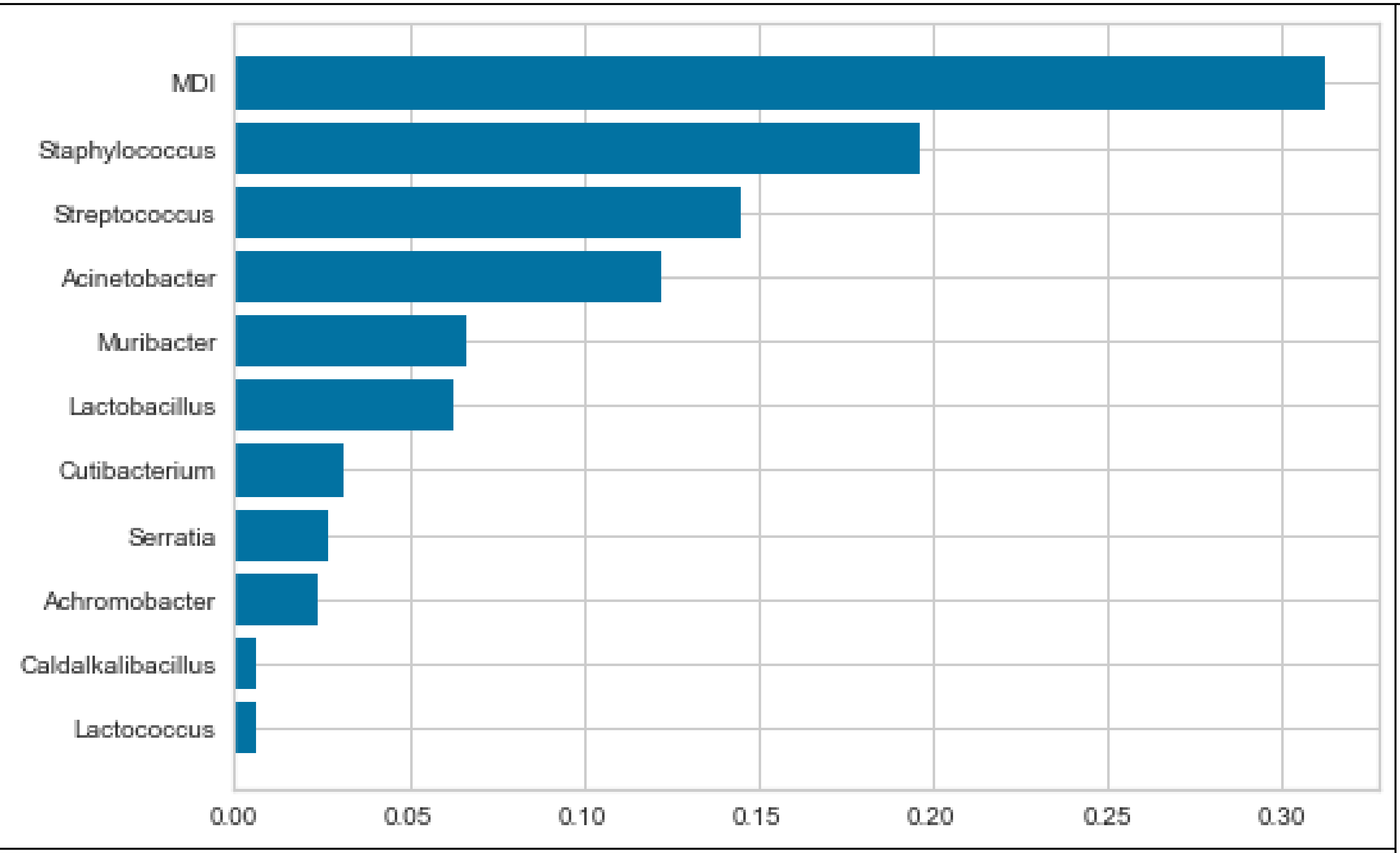

A

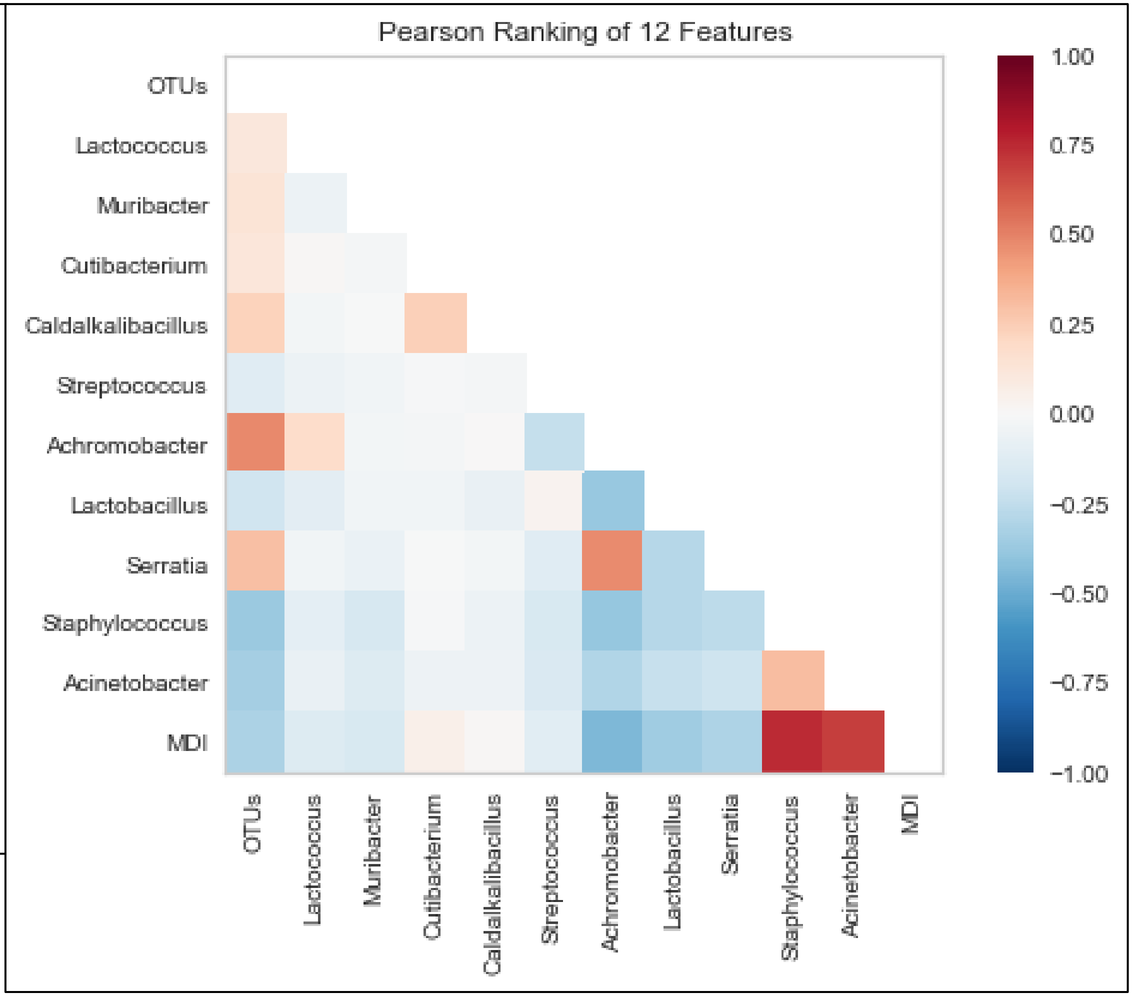

B

TABLE S1. The peptide sequences of three AMPs and their corresponding gAMPs with multimerin1 fragment fused to the N-terminus separated by a linker

AMP/ gAMP	Peptide Sequence
Laterosporulin	ACQCPDAISGWTHTDYQCHGLENKMYRHVYAICMNGTQVYCRTEWGSSC
Multimerin1(MM1)-laterosporulin	MQKMTDQVNYQAMKLTLLQKSGGGSACQCPDAISGWTHTDYQCHGLENKMYRHVYAICMNGTQVYCRTEWGSSC
Alyteserin	GLKDIFKAGLGSLVKGIAAHVAN
MM1-alyteserin	MQKMTDQVNYQAMKLTLLQKSGGGSGLKDIFKAGLGSLVKGIAAHVAN
CRAMP	ISRLAGLLRKGGEKIGEKLKKIGQKIKNFFQKLVPQPE
MM1-CRAMP	MQKMTDQVNYQAMKLTLLQKSGGGSISRLAGLLRKGGEKIGEKLKKIGQKIKNFFQKLVPQPE

Blue = Multimerin1, Red = linker

Table S2. p-values of all CFU values of *H. pylori* for all treatment vs Day for in vivo therapeutic experiments

Day	pTKR	Alyteserin	MM1-Alyteserin	CRAMP	MM1-CRAMP	Laterosporulin	MM1-Laterosporulin	Antibiotic	Treatment
0	0.34607	0.467459	0.215381	0.081516	0.254538184	0.776073	0.103358	0.387186	**Null**
5	0.015451	0.428519	0.087484	0.975067	0.870429205	0.444576	0.267252	0.198333	
8	0.008943	0.007684	0.007411	0.007414	0.007411531	0.008125	0.007705	0.030924	
10	0.005192	0.003831	0.003807	0.003802	0.003770856	0.003895	0.003842	0.00427	
0	1	0.176318	0.111203	0.291126	0.13263648	0.091847	0.231938	0.454293	**pTKR**
5	1	0.01587	0.007915	0.017646	0.002896649	0.061365	0.042453	0.057843	
8	1	0.07307	0.053533	0.052685	0.052660441	0.190541	0.075258	0.062701	
10	1	0.039988	0.038568	0.038282	0.036621635	0.043789	0.040658	0.081771	
0		1	0.285696	0.083002	0.32489647	0.229294	0.119879	0.451168	**Alyteserin**
5		1	0.053075	0.228006	0.241878544	0.111932	0.08035	0.142134	
8		1	0.184734	0.043186	0.079155408	0.147012	0.451542	0.049455	
10		1	0.200829	0.125313	0.020322686	0.039267	0.380268	0.081643	
0			1	0.100896	0.460872819	0.154003	0.183151	0.314591	**MM1-Alyteserin**
5			1	0.106988	0.030519649	0.322362	0.368682	0.07015	
8			1	0.433618	0.453738191	0.087472	0.170367	0.046996	
10			1	0.394445	0.047280591	0.005988	0.165983	0.077365	
0				1	0.122830099	0.08951	0.185735	0.107199	**CRAMP**
5				1	0.437479122	0.263403	0.190216	0.096216	
8				1	0.47311071	0.073903	0.049111	0.04701	
10				1	0.010066071	0.003126	0.120219	0.07658	
0					1	0.165327	0.194758	0.358203	**MM1-CRAMP**
5					1	0.189996	0.107719	0.103198	
8					1	0.076802	0.077756	0.046989	
10					1	0.001084	0.032913	0.071742	
0						1	0.10422	0.207353	**Laterosporulin**
5						1	0.432257	0.074787	
8						1	0.156808	0.054122	
10						1	0.115616	0.0939	
0							1	0.13775	**MM1-Laterosporulin**
5							1	0.072487	
8							1	0.049649	
10							1	0.083609	

Table S3. CFU values of *H. pylori* for all treatment vs Day for in vivo therapeutic experiments

**log10(CFU/ml)**	**Day**	**Null**		**Empty *L. lactis***		**Alyteserin**		**MM1-Alyteserin**		**CRAMP**		**MM1-CRAMP**		**Laterosporulin**		**MM1-Laterosporulin**		**Antibiotic**	
		**Mean**	**SE**	**Mean**	**SE**	**Mean**	**SE**	**Mean**	**SE**	**Mean**	**SE**	**Mean**	**SE**	**Mean**	**SE**	**Mean**	**SE**	**Mean**	**SE**
	0	4.168055	1.339393	4.498902	1.270948	3.873131	1.293921	3.653439	1.272044	3.713066	1.175893	4.085446	1.238243	4.081421	1.323419	3.582956	1.191635793	4.728116	1.239987
	5	6.647503	1.217981	6.011062	1.187677	6.887585	1.137737	6.844684	1.129154	7.024468	1.171769	6.904182	1.103229	6.632587	1.224588	6.767043	1.140779031	7.432851	1.15541
	8	7.187798	1.079283	6.240183	1.235639	5.22933	1.265512	5.554992	1.182607	5.812495	1.134802	5.680376	1.142421	6.141252	1.144018	5.938671	1.143877798	6.759111	1.111848
	10	7.607933	1.135349	7.192776	1.334218	4.446757	1.317031	4.195873	1.250462	5.016954	1.22951	4.59946	1.246162	4.900758	1.268642	4.378478	1.336920645	6.136341	1.237059

**CFU/ml**	**Day**	**Null**		**Empty *L. lactis***		**Alyteserin**		**MM1-Alyteserin**		**CRAMP**		**MM1-CRAMP**		**Laterosporulin**		**MM1-Laterosporulin**		**Antibiotic**	
		**Mean**	**SE**	**Mean**	**SE**	**Mean**	**SE**	**Mean**	**SE**	**Mean**	**SE**	**Mean**	**SE**	**Mean**	**SE**	**Mean**	**SE**	**Mean**	**SE**
	0	92333.91	42245.58	89668.4	28335.72	58214.05	33316.33	34940.07	20761.93	15186.72	10296.03	44454.95	25015.82	124031.9	75841.6	12789.27	8570.862797	78258.8	33236.77
	5	8218373	2066538	2370862	940991.6	10941435	3958493	10100926	2837459	16562757	4161146	10480457	2391655	10462486	3274822	8875687	2025373.97	31985201	9157343
	8	17133780	2258701	16199934	14157292	370209.5	89734.34	891391.2	356773.2	1220568	536374.6	801466.2	224198.5	2073070	681775.2	1770736	706726.3799	6341958	1624273
	10	71762618	37710926	1.55E+08	95564168	143289.8	43839.5	33472.23	31651.96	216570	50234.84	97636.05	43320.11	230020.8	58140.26	119827	55929.91934	1910994	657413.1

Table S4. p-values between observed OTUs for all therapeutic treatment regimen amongst different days of experiment

Days	AMP-*L. lactis*				gAMP-*L. lactis*				Antibiotic				Empty-*L. lactis*				Only *H. pylori*			
	**0**	**5**	**8**	**10**	**0**	**5**	**8**	**10**	**0**	**5**	**8**	**10**	**0**	**5**	**8**	**10**	**0**	**5**	**8**	**10**
**0**	1.00000	0.00001	0.00018	0.46100	1.00000	0.00000	0.00000	0.52660	1.00000	0.03789	0.22917	0.17932	1.00000	0.00349	0.00352	0.98184	1.00000	0.00244	0.00108	0.00001
**5**		1.00000	0.01810	0.00000		1.00000	0.04781	0.00000		1.00000	0.00332	0.00061		1.00000	0.84182	0.00895		1.00000	0.72215	0.01893
**8**			1.00000	0.00000			1.00000	0.00000			1.00000	0.02482			1.00000	0.01452			1.00000	0.02063
**10**				1.00000				1.00000				1.00000				1.00000				1.00000

Table S5. p-values between observed OTUs for all therapeutic treatment regimen amongst themselves at each day of the experiment

	AMP-L.lactis	gAMP-L.lactis	Antibiotic	Empty-L.lactis	Days
**gAMP-*L. lactis***	0.026854613				0
	0.081931714				5
	0.703164546				8
	0.117640308				10
**Antibiotic**	0.987968122	0.360921299			0
	0.001742615	0.003148531			5
	0.155909767	0.074791408			8
	4.93273E-08	7.71292E-08			10
**Empty-*L.lactis***	0.026854613	0.653996412	0.48317939		0
	0.081931714	0.206861429	0.51444783		5
	0.0703164546	0.0745047107	0.01940923		8
	0.1933831747	0.0117640308	0.0071271		10
H. pylori	0.044265569	0.320987335	0.54201676	0.769715646	0
	0.033777062	0.043089091	0.34828784	0.230365208	5
	0.067260472	0.098598758	0.03550178	0.135036551	8
	2.91726E-06	4.66188E-07	0.25261204	0.005933432	10

Table S6. Observed OTUs for all therapeutic treatment regimens for each day of the experiment

Treatment	Day	Mean	SE
**AMP-*L. lactis***	0	111.551077	12.40330351
	5	38.0295739	1.73867928
	8	51.7975174	4.849706376
	10	123.06498	8.434694901
**gAMP-*L. lactis***	0	160.212938	16.6492163
	5	42.7670679	1.894378149
	8	54.5856944	5.071584638
	10	166.963241	11.47240435
**Antibiotic**	0	112.259259	33.06344126
	5	66.8833333	3.340274024
	8	43.0791667	2.645458471
	10	30.1666667	2.190044816
**Empty-*L. lactis***	0	148.238804	18.4746069
	5	58.9169841	9.557273309
	8	56.5755556	2.573158097
	10	148.879167	16.13032121
H. pylori	0	141.869444	5.50675984
	5	76.6513889	6.443820711
	8	72.6178836	5.728003074
	10	40.118	6.45212052

Table S7. p-values between observed OTUs for all prophylactic treatment regimen amongst different days of experiment

**Day**	**AMP-*L. lactis***				**gAMP-*L. lactis***				**Empty-*L. lactis***				***H. pylori***		
	**0**	**3**	**8**	**10**	**0**	**3**	**8**	**10**	**0**	**3**	**8**	**10**	**3**	**8**	**10**
**0**	1.00000	0.01009	0.84404	0.04680	1.00000	0.00031	0.52342	0.04768	1.00000	0.86998	0.00665	0.57011			
**3**		1.00000	0.00144	0.00007		1.00000	0.00197	0.23669		1.00000	0.00001	0.52462	1.00000	0.00208	0.00009
**8**			1.00000	0.00510			1.00000	0.03520			1.00000	0.00295		1.00000	0.00209
**10**				1.00000				1.00000				1.00000			1.00000

Table S8. p-values between observed OTUs for all prophylactic treatment regimen amongst themselves at each day of the experiment

	**AMP-*L. lactis***	**gAMP-*L. lactis***	**Empty-*L. lactis***	**Days**
**gAMP-*L. lactis***	0.931305566			0
	0.228948433			3
	0.632562178			8
	0.013133248			10
**Empty-*L. lactis***	0.477512806	0.366176625		0
	0.008350127	0.004004745		3
	0.000311906	0.004527501		8
	0.022369944	0.0060257183		10
H. pylori	0.056975984	0.191648431	0.330431167	3
	0.007091466	0.077699665	0.019776596	8
	0.00552311	0.004658643	0.000802383	10

Table S9. Observed OTUs for all prophylactic treatment regimens for each day of the experiment

Treatment	Day	Mean	SE
**AMP-*L. lactis***	0	103.3175926	14.59940328
	3	168.3703704	11.39806636
	8	99.64814815	7.577382989
	10	61.09206349	6.534697096
**Empty-*L. lactis***	0	121.9798942	17.83894003
	3	118.4648148	5.920348311
	8	39.95376984	7.33390052
	10	107.3444444	14.51688127
**gAMP-*L. lactis***	0	101.7555556	6.416933616
	3	150.2619048	5.343384735
	8	92.2375	11.78199331
	10	205.4111111	38.63256452
H. pylori	3	131.9488889	9.906642042
	8	65.48302154	4.224867978
	10	24.95	6.486694073

Table S10. Species specific primers used in the qPCR quantification analysis

**Target bacteria**	**Target gene/protein**	**Sequence**
H. pylori	*vacA*	forward: 5′-ATGGAAATACAACAAACACAC-3′;<br>reverse: 5′-CTGCTTGAATGCGCCAAAC-3′
L. lactis	*acmA*	forward: 5’-GGAGCTCGTGAAAGCTGACT-3’;<br>reverse: 5’-GCCGGAACATTGACAACCAC-3’
E. coli	DE3-T7 polymerase	forward: 5’-GTGTGGGCACACGAGAAGTA-3’;<br>reverse: 5’-GAGACTCGTGCAACTGGTCA-3’
L. plantarum	*recA*	forward: 5′-CCGTTTATGCGGAACACCTA-3′;<br>reverse: 5′-TCGGGATTACCAAACATCAC-3′
